# UNRAVELING CRP/cAMP-MEDIATED METABOLIC REGULATION IN *ESCHERICHIA COLI* PERSISTER CELLS

**DOI:** 10.1101/2024.06.10.598332

**Published:** 2024-06-10

**Authors:** Han G. Ngo, Sayed Golam Mohiuddin, Aina Ananda, Mehmet A. Orman

**Affiliations:** 1 Department of Chemical and Biomolecular Engineering, University of Houston, TX, 77204; 2 Department of Biology, Monmouth University, NJ, 07764

## Abstract

A substantial gap persists in our comprehension of how bacterial metabolism undergoes rewiring during the transition to a persistent state. Also, it remains unclear which metabolic mechanisms become indispensable for persister cell survival. To address these questions, we directed our efforts towards persister cells in *Escherichia coli* that emerge during the late stationary phase. These cells have been recognized for their exceptional resilience and are commonly believed to be in a dormant state. Our results demonstrate that the global metabolic regulator Crp/cAMP redirects the metabolism of these antibiotic-tolerant cells from anabolism to oxidative phosphorylation. Although our data indicates that persisters exhibit a reduced metabolic rate compared to rapidly growing exponential-phase cells, their survival still relies on energy metabolism. Extensive genomic-level analyses of metabolomics, proteomics, and single-gene deletions consistently emphasize the critical role of energy metabolism, specifically the tricarboxylic acid (TCA) cycle, electron transport chain (ETC), and ATP synthase, in sustaining the viability of persisters. Altogether, this study provides much-needed clarification regarding the role of energy metabolism in antibiotic tolerance and highlights the importance of using a multipronged approach at the genomic level to obtain a broader picture of the metabolic state of persister cells.

## INTRODUCTION

Bacterial persisters within cell cultures constitute a small subpopulation of cells exhibiting a transient antibiotic-tolerant state ^1^. While persisters have traditionally been characterized as non-growing and dormant phenotypes ^2^, recent studies challenge these conventional hallmarks, revealing the heterogeneity of persister cells in terms of growth, metabolism, and other cellular activities ^3-11^. Despite potential discrepancies in research outcomes in the field, we think that these variations arise from the intricate and diverse survival mechanisms employed by bacterial cells in response to adverse conditions, such as antibiotic treatments. Furthermore, the interplay of stochastic and deterministic factors associated with these mechanisms adds another layer of complexity, with outcomes highly contingent on factors such as cell types, antibiotics, and experimental and growth conditions ^12^. The persistence phenomenon presents a significant health concern ^13,14^, as the transient antibiotic-tolerant state of persister cells promotes recurrent infections ^15^ and establishes them as a reservoir for the emergence of antibiotic-resistant mutants ^16-18^.

Drug tolerance is a widespread phenomenon observed in both prokaryotic and eukaryotic cell types. Otto Warburg's research in the early twentieth century unveiled an intriguing aspect of mammalian cell metabolism known as "aerobic glycolysis," wherein proliferating cells (e.g., tumor cells) derive energy predominantly through glycolysis, even in the presence of oxygen ^19^. This metabolic reprogramming involves restricting entry into the TCA cycle through precise enzymatic control, diverting glycolytic intermediates towards anabolic pathways. This adaptation supports the extensive biosynthesis required for active cell proliferation in tumors ^20^. Remarkably, tumorigenic persisters that exist in a non-proliferating state may not primarily depend on aerobic glycolysis. Instead, there is substantial evidence suggesting that these cells rely on energy metabolism ^21,22^; however, the presence of this metabolic state in antibiotic-tolerant bacteria is still a question mark ^10^. Reprogramming energy metabolism seems to be an evolutionarily conserved strategy for cells facing stress or adverse conditions, as these cells might benefit from the significantly higher ATP production efficiency provided by oxidative phosphorylation ^20^. The identification of a mechanism shared by diverse cell types could open avenues for the development of global strategies to target drug-tolerant cells effectively.

A recent study suggests that bacterial persisters constitute a stochastically formed subpopulation of low-energy cells, despite some observed overlap in ATP levels between antibiotic-sensitive and persister cells ^23^. The persister cells examined in that study were derived from an aged stationary phase culture (48 hours post-inoculation) ^23^, a condition known to elevate the number of non-growing cells, which do not promptly resume growth upon transfer to a fresh medium ^24^. While the non-growing cells formed during the stationary phase have reduced metabolic activity compared to growing cells, as demonstrated in our earlier study ^11^, they still exhibit a certain degree of respiratory activity when their metabolism was characterized within the stationary phase culture ^10^. We found that although there were significantly more persister cells in this non-growing cell subset, persister cells were still present in the growing cell populations ^11^. This finding also aligns with another independent study, which utilized single-cell analysis, reporting that ofloxacin persisters were metabolically active cells in exponentially growing cultures before treatment and these cultures were obtained from 16-hour overnight precultures (not aged) ^25^. The nature of persister-cell metabolism in bacteria is a topic that has long been a point of contention in scientific circles. The controversy surrounding this topic primarily arises from studies that rely on bacteriostatic chemicals or a limited number of gene deletions, or direct comparisons to exponentially growing cells, all of which have inherent drawbacks ^10,11,23,26^. The metabolism of persister cells, a very complex phenomenon, cannot be easily characterized by a simplistic term such as “metabolic dormancy”. Even if persister cells may exhibit a lower metabolism compared to the vast majority of rapidly growing exponential-phase cells, they may still rely on energy metabolism for their functioning ^27,28^. In fact, analyzing published studies collectively suggests that bacterial cell metabolism undergoes intricate alterations or rewiring as they transition into a tolerant state, and these alterations seem to be highly dependent on the specific conditions tested ^3-7,9-11^.

To gain a better understanding of the critical role of energy metabolism in persister cell survival, we have focused our research on antibiotic-tolerant cells formed during the stationary phase, given that these cells are known to be highly resilient and capable of surviving a variety of stressors, including antibiotics, and are assumed to be dormant ^1,10^. These cells are also referred to as type I persisters, which cannot readily resume growth when diluted in fresh media during the lag phase ^1^. Previous studies showed that these persister cells can metabolize specific carbon sources that make them susceptible to aminoglycosides (AG) ^29-31^. Their AG susceptibility is due to increased AG uptake, which is facilitated by increased ETC activity and membrane potential ^29^. The presence of active energy metabolism in antibiotic-tolerant, non-growing cells may indeed explain their rapid killing by AG in the presence of carbon sources ^10,30^. When the knockout strains of global transcriptional regulators (i.e., ArcA, Cra, Crp, DksA, Fnr, Lrp, and RpoS) were screened using the AG potentiation assay in our previous study, the results showed that the panel of carbon sources tested potentiated the AG killing of tolerant cells derived from most knockout strains, except for Δ*crp* and Δ*cyaA* ^31^. This can be attributed to the lack of active energy metabolism in these mutant strains, as the Crp/cAMP potentially shapes persister cell metabolism during the stationary phase. Depletion of primary carbon sources activates adenylate cyclase (CyaA) ^32^, increasing cyclic-AMP (cAMP) levels in cells ^33,34^. The cAMP molecules, along with its receptor protein (Crp), activate genes related to the catabolism of secondary carbon sources, potentially supporting cellular functions and energy levels ^35-38^. Here, using metabolomics, proteomics, and high-throughput screening of single-gene deletion strains, we have provided evidence that the Crp/cAMP regulatory complex maintains an active state of energy metabolism while downregulating anabolic pathways in the antibiotic-tolerant persister cells.

## RESULTS

### Disruption of the Crp/cAMP complex affects the formation of persister cells at the late stationary phase.

**Figure 1:**
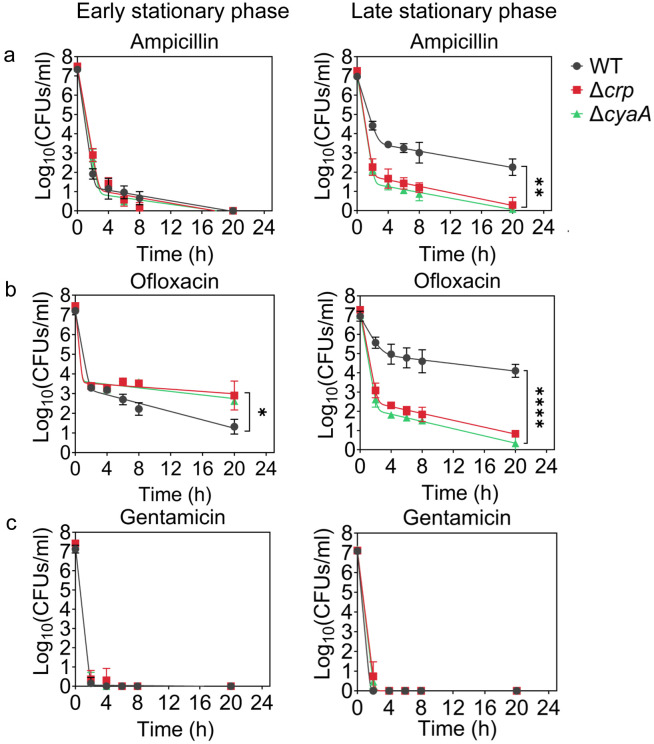
Crp/cAMP Regulation of Persister Cell Formation in the Stationary Phase. *E. coli* K-12 MG1655 WT and mutant cells at early (t=5h) and late (t=24h) stationary phases were transferred to fresh medium with antibiotics for persister cell quantification. At time points 0, 2, 6, 8, and 20h, 1 mL of the treated culture underwent two washes with 1X phosphate-buffered saline (PBS) to remove antibiotics. It was then serially diluted and plated on an agar plate to count the colony-forming units (CFUs). **(a)** Persister levels of ampicillin-treated culture with an antibiotic concentration of 200 μg/mL. **(b)** Persister levels of ofloxacin-treated culture with an antibiotic concentration of 5 μg/mL. **(c)** Persister levels of gentamicin-treated culture with an antibiotic concentration of 50 μg/mL. The number of biological replicates is n=4 for all panels. Biphasic kill curves were generated using a non-linear model (see Materials and Methods). Statistical significance tests were conducted using F-statistics (*P < 0.05, **P < 0.01, ****P < 0.0001). The data for each time point represent the mean value ± standard deviation.

Since Crp/cAMP-mediated metabolic changes can be induced by nutrient depletion during the stationary phase, we wanted to assess the effects of deleting the *crp* and *cyaA* genes (Δ*crp* and Δ*cyaA*) on both persister cell formation and metabolism during this phase. As anticipated, the deletion of the *cyaA* gene resulted in a notable reduction in intracellular cAMP concentration (Supplementary Fig. 1). However, the Δ*crp* strain exhibited an increase in cAMP concentration, potentially due to the negative feedback regulatory mechanism of the Crp/cAMP complex for the *cyaA* gene promoter ^39,40^. When comparing the growth curves of *E. coli* wild-type (WT), Δ*crp*, and Δ*cyaA* main cultures under identical conditions studied here (see Materials and Methods), all three strains started to enter the stationary phase around 5 hours, with the mutant strains exhibiting slightly lower optical density levels than the WT at this time point **(Supplementary Fig. 2).** Also, our data provide evidence of an increase in cAMP levels in WT cells during their transition into the stationary phase **(Supplementary Fig. 3),** aligning with existing literature ^33,34,40^. For type I persister quantification, we diluted cells in the fresh medium (consistent with previous studies ^1,10^) at early (t=5h) and late (t=24h) stationary phases and subsequently exposed them to an extended period (20 hours) of ampicillin or ofloxacin treatment (200 μg/mL ampicillin and 5 μg/mL ofloxacin). These treatments were carried out at concentrations surpassing the minimum inhibitory concentrations (MIC), necessary for the selection of antibiotic-tolerant persister cells **(Supplementary Table 1)** ^41^. Also, type I persisters, formed during the stationary phase, exhibit a slow transition from a non-growing state to an active state when transferred to a fresh medium in the lag phase ^1,10,42,43^; therefore, this transition requires longer antibiotic treatment durations. Moreover, we transferred an equal number of cells from each strain to the fresh medium to ensure consistency in cell numbers. Our findings revealed biphasic kill curves, indicative of persistence phenotypes, with the WT strain showing a notable increase in both ampicillin and ofloxacin-persister cells at the late stationary phase, in contrast to the mutant strains where no such increase was observed (Fig. 1a,b). However, no such trend was reported in the early stationary phase (Fig. 1a,b). To confirm that the observed decrease in persister levels in the mutant strains in the late stationary phase is solely attributed to the perturbation of the Crp/cAMP regulatory network, we reintroduced *crp* expression to the Δ*crp* strain using a low-copy plasmid carrying the *crp* gene and its promoter. As a control, we utilized an empty vector of the same plasmid. The results demonstrated that the expression of *crp* restored the persister level in the mutant strain, while the plasmid itself had no impact on persister levels **(Supplementary Fig. 4a,b).** Altogether, these findings highlight the significant role of Crp/cAMP in ampicillin and ofloxacin persister formation in the late stationary phase.

Ampicillin and ofloxacin, both broad-spectrum antibiotics with a strong dependence on cell metabolism ^44^, target cell wall synthesis and DNA gyrase activity, respectively. In addition to these two antibiotics, we also quantified aminoglycoside-persister levels in both WT and mutant strains. This was achieved by exposing diluted cells from both early and late stationary phases to 50 μg/mL gentamicin, a concentration exceeding the MIC levels **(Supplementary Table 1).** After prolonged gentamicin exposure (20 h), the tolerant cell colonies were found to be below the limit of detection for all strains and conditions (Fig. 1c, and **Supplementary Fig. 4c**). Although bacterial tolerance can vary significantly depending on the specific antibiotics and growth phase used ^45^, this outcome contrasts starkly with the observed levels of ampicillin and ofloxacin persisters in WT (Fig. 1). The mechanism by which aminoglycosides eliminate persister cells in the WT strain may be linked to their metabolism, given that aminoglycoside uptake is an energy-requiring process ^29,46^, and this aspect will be explored further in the subsequent section.

### Crp/cAMP complex governs *E. coli* stationary phase metabolism.

**Figure 2:**
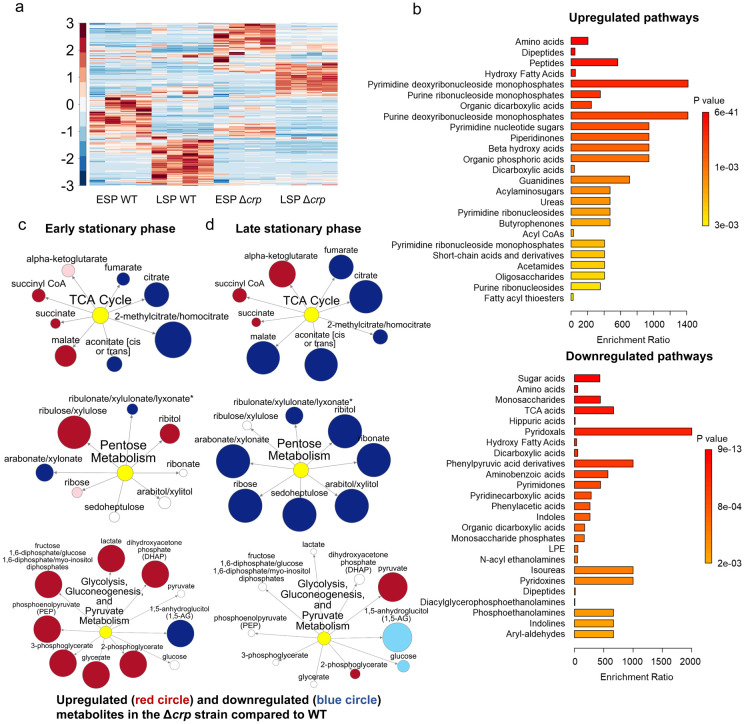
The effect of Crp/cAMP on Persister Cell Metabolism during Stationary Phase. **(a)** MS analysis of *E. coli* K-12 MG1655 WT, Δ*crp*, and Δ*cyaA* at early (t=5h) and late (t=24h) stationary phases. Unsupervised hierarchical clustering was applied to standardized metabolic data. Each column represents a biological replicate. n=4. **(b)** Pathway enrichment analysis was conducted using MetaboAnalyst^47^. Upregulated and downregulated pathways of the Δ*crp* strain compared to WT in the late-stationary growth phase were provided in this figure (refer to Supplementary Fig. 5, 6, and 7 for the other pairwise comparisons). **(c, d)** Pathway enrichment maps comparing metabolites of the TCA cycle, pentose phosphate metabolism, glycolysis, gluconeogenesis, and pyruvate metabolism in Δ*crp* versus WT for ESP and LSP conditions, respectively. Circle size corresponds to the ratio of normalized metabolite intensities between mutant and control cells. Blue (P ≤ 0.05 for dark blue; 0.05 < P < 0.10 for light blue) and red (P ≤ 0.05 for dark red; 0.05 < P < 0.10 for light red) indicate significantly downregulated or upregulated metabolites in the mutant compared to the control. White signifies no significant difference. n=4 for all panels. ESP: Early stationary phase, LSP: Late stationary phase.

To determine whether the reduced ampicillin and ofloxacin persister levels at the late stationary phase in the mutant strains (Fig. 1a,b) are linked to stationary phase metabolism, we utilized untargeted mass spectrometry (MS). This approach facilitated the quantification of metabolites in Δ*crp* cells, allowing for a comparison with WT controls in both the early and late stationary phases. (Fig. 2a). The metabolomics data were subjected to unsupervised hierarchical clustering, and metabolites identified in independent biological replicates of each strain and condition were found to cluster together (Fig. 2a), thus confirming the reproducibility of our data.

To elucidate the upregulated and downregulated metabolic pathways in the mutant strain as compared to the WT strain, we performed enrichment analyses utilizing MetaboAnalyst ^47^. For downregulated pathways, we considered a threshold ratio of 0.5 or lower, where the ratio indicates metabolite levels in the mutant strain relative to the WT **(Supplementary Table 2).** Conversely, for upregulated pathways, the threshold ratio was set at 2 or higher **(Supplementary Table 3).** The enrichment ratio for each pathway was calculated based on the number of metabolite hits compared to the expected hits derived from the chemical structure library ^47^. Our extensive comparison of the mutant cells to the WT cells through pathway enrichment analysis (refer to Fig. 2b and **Supplementary Fig. 5, 6 and 7** for pairwise comparison of different conditions) revealed several important findings:

**(i)** During the early stationary phase, we observe a slight downregulation in the abundance of TCA cycle metabolites, including citrate and fumarate, in the Δ*crp* strain compared to WT (Fig. 2c and **Supplementary Fig. 5).** However, as the late stationary phase progresses, the downregulation in both TCA cycle and pentose phosphate metabolism becomes more pronounced in the Δ*crp* strain (Fig. 2b, d).

**(ii)** The Δ*crp* strain exhibits upregulation of several metabolites compared to WT during both early and late stationary phases, primarily associated with anabolic pathways. Particularly, the upregulation of some of these pathways becomes more pronounced during the late stationary phase. These pathways include crucial metabolites like deoxyribonucleosides and ribonucleosides (which play essential roles in DNA and RNA synthesis), fatty acids and carboxylic acids (the main components of bacterial cell membranes), and peptides (which are linked to protein synthesis) (Fig. 2b).

**(iii)** During the early stationary phase, we noticed a significant upregulation in the abundance of intermediate metabolites related to glycolysis, gluconeogenesis, and pyruvate metabolism in mutant cells compared to WT cells (Fig. 2c). This observation is not surprising, as the inhibition of the TCA cycle in the mutant strain could potentially redirect metabolic fluxes toward glycolysis and lactate metabolism.

Altogether, our metabolic data indicate that, in the stationary phase, WT cells maintain their energy metabolism to some extent while downregulating their anabolic pathways (Fig. 2a). This metabolic state appears to be regulated by the Crp/cAMP complex, as perturbing its function leads to a significant downregulation of energy metabolism and an upregulation in the abundance of anabolic metabolites (Fig. 2b).

### Proteomics analysis revealed upregulated pathways in the Δ*crp* strain associated with anabolic metabolism, alongside the downregulation of key proteins in energy metabolism.

**Figure 3:**
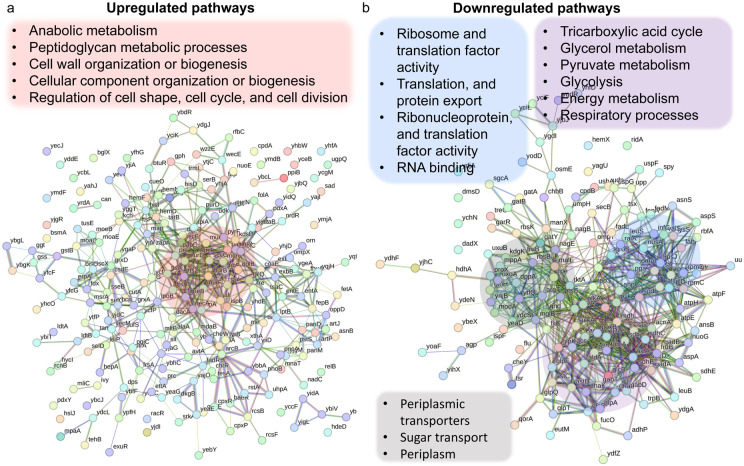
Validation of Crp/cAMP-Mediated Metabolic State in Persister Cells Through Proteomics Analysis. Pathway enrichment analysis was conducted in STRING ^48,49^ for upregulated **(panel a)** and downregulated **(panel b)** proteins. Genes highlighted in red are linked with the upregulated protein networks, while genes in blue, gray, and purple correspond to those in the downregulated protein network. The visual network in STRING illustrates protein interactions. In evidence mode, color in the network represents the interaction evidence of data support, derived from curated databases, experimental data, gene neighborhood, gene fusions, co-occurrence, co-expression, protein homology, and text mining ^48,49^.

Since the Crp/cAMP complex acts as a transcriptional regulator affecting the expression of metabolic proteins whose abundance directly affects cellular metabolites, we performed untargeted proteomics, our second genomic-level study, to further validate our results. Considering the noticeable metabolic alterations observed during the late stationary phase, we utilized MS to quantify proteins in Δ*crp* cells and compared them to WT controls at this stage. The resulting proteomics data were subjected to unsupervised hierarchical clustering, and the proteins identified in independent biological replicates of each strain were found to cluster together, confirming the consistency of our findings **(Supplementary Fig. 8).** By analyzing protein-protein association networks and employing functional enrichment through STRING ^48,49^, which integrates various functional pathway classification frameworks such as Gene Ontology annotations, KEGG pathways, and UniProt keywords, we pinpointed various upregulated and downregulated pathways in the mutant strain when compared to the WT **(Supplementary Tables 4 and 5).** The upregulated pathways are associated with anabolic metabolism and encompass peptidoglycan metabolic processes, cell wall organization or biogenesis, cellular component organization or biogenesis, regulation of cell shape, cell cycle, and cell division (Fig. 3a). Also, the mutant strain displayed downregulated pathways, encompassing glycerol metabolism, TCA cycle, pyruvate metabolism, glycolysis, and various pathways associated with ribosome and transcriptional factor activity (Fig. 3b). Our analysis specifically pinpointed a cluster of proteins involved in energy metabolism and respiratory processes. Notably, this cluster includes GltA, SdhB, SucC, SucD, FrdB, FrdA, AcnA, AceA, and Mdh proteins, which play crucial roles in either the TCA cycle or as membrane-bound components of ETC (Fig. 3b). Altogether, the alignment between metabolomics and proteomics analyses provides additional validation for the Crp/cAMP-mediated metabolic state.

### Crp/cAMP complex shapes *E. coli* cell proliferation dynamics.

**Figure 4:**
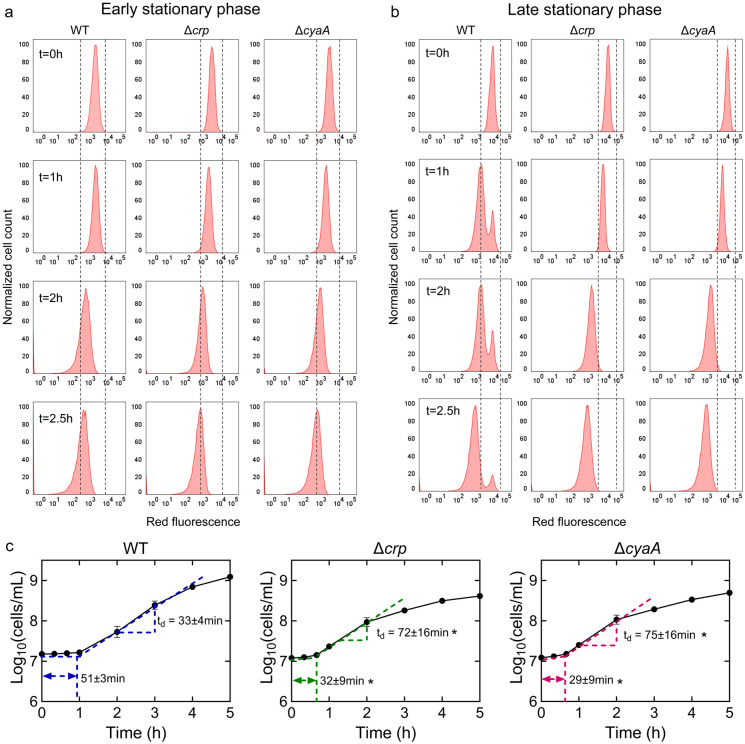
The Role of Crp/cAMP in Non-Growing Cell Formation. **(a, b)** Flow cytometry histograms depict mCherry expression in *E. coli* K-12 MG1655 WT, Δ*crp*, and Δ*cyaA* at early (t=5h) and late (t=24h) stationary phases, respectively. Cells containing an IPTG-inducible mCherry expression system were cultivated with IPTG. After washing and dilution of early and late stationary phase cells in IPTG-free fresh media, fluorescence was tracked in non-growing and growing cells for 2.5 hours. The panel is a representative biological replicate. Consistent results were seen across all 3 biological replicates. **(c)** Growth curves of WT, Δ*crp*, and Δ*cyaA* cultures were determined using flow cytometry to calculate lag and doubling times. Lag times were calculated using the "Microbial lag phase duration calculator" ^79^. Doubling times were computed using the formula t_d_=Δt/(3.3xLog_10_(N/N_o_)). n=3. *Statistical significance observed between control and mutant strains (P < 0.05, 2-tailed t-test). The data for each time point represent the mean value ± standard deviation.

The omics data suggest that the upregulation in the abundance of anabolic metabolites and proteins, particularly those related to cell wall organization or biogenesis, cell cycle, and cell division, in the stationary-phase mutant cells, would likely enhance their ability to resume growth upon transitioning to fresh medium. To investigate this, we utilized a cell proliferation assay that employed an inducible fluorescent protein (mCherry) expression cassette. This assay provided us with the ability to monitor non-growing cells at a single-cell resolution, as described previously ^11,50^. The mCherry expression cassette is controlled by an isopropyl ß-D-1-thiogalactopyranoside (IPTG) inducible synthetic T5 promoter that was previously inserted into the chromosome of an *E. coli* strain carrying a *lacI*^*q*^ promoter mutation ^11^. This configuration allowed for precise regulation of mCherry expression using IPTG. Here, we introduced *crp* and *cyaA* deletions into this strain. These deletions reduced the persistence of the mCherry-expressing *E. coli* strain in the late stationary phase **(Supplementary Fig. 9),** consistent with the findings presented in Fig. 1. To perform the growth assay, we induced mCherry expression in the main cultures and then washed the cells to remove IPTG. The cells were then inoculated into a fresh medium without IPTG and their growth was analyzed with a flow cytometry. This allowed us to track the dilution of mCherry protein within the cells, which served as an indicator of cell proliferation. As shown in Fig. 4a, initially, all cells exhibited high red fluorescence. However, as cells underwent division, the red fluorescence of the overall population decreased in the absence of the inducer (Fig. 4a). Notably, within the WT strain, a subpopulation from the late stationary phase cultures displayed constant fluorescence levels, indicating their inability to divide (Fig. 4b). In contrast to the WT strain, we did not detect similar subpopulations in the mutant strains (Fig. 4b). Additionally, this subpopulation of non-growing cells does not emerge during the early stationary phase cultures (Fig. 4a). This observation provides an explanation for the observed reduction in persister levels in these mutant strains in the late stationary phase, as the enrichment of persister cells within these non-growing cell subpopulations was reported in previous studies ^30,50,51^.

To confirm the significance of Crp/cAMP in the formation of non-growing cells, we introduced the expression plasmid carrying the *crp* gene into the Δ*crp* strain. As anticipated, the introduction of the *crp* expression plasmid resulted in the emergence of a non-growing population within the culture, contrasting with the mutant strain containing the empty plasmid vector used as a control **(Supplementary Fig. 10a, b).** The reduced capacity of stationary-phase WT cells to initiate proliferation upon transfer to a fresh medium suggests the possible presence of an extended lag phase in these cells. To investigate this, we employed flow cytometry to precisely quantify cell numbers and generate growth curves for both WT and mutant strains. As anticipated, the growth curve of the WT strain displayed a slower initial growth rate and a prolonged lag phase duration compared to the mutant strains (Fig. 4c).

Conversely, the mutant strains displayed a shorter lag phase, yet they demonstrated an increased doubling time in the exponential phase compared to WT (Fig. 4c), which was also anticipated, considering their decreased reliance on oxidative phosphorylation due to TCA cycle inhibition. Altogether, these results provide additional support and validation for the findings from our metabolomics and proteomics data, as our data reveals a correlation between the abundance of molecules associated with cell division and the ability of the stationary phase cells to resume growth.

### Persister cells rely on energy metabolism.

**Figure 5:**
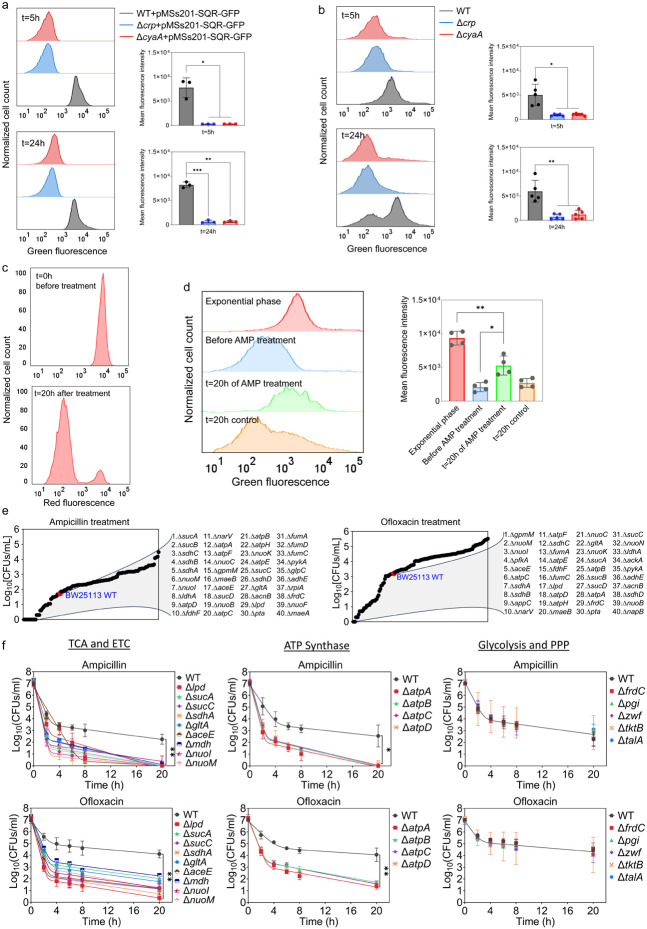
Crp/cAMP-Mediated Metabolic State of Persister Cells. **(a)** GFP reporter plasmid introduced into *E. coli* K-12 MG1655 WT, Δ*crp*, and Δ*cyaA* cells to monitor SQR gene activity. Flow cytometry was used to detect activity at early (t=5h) and late (t=24h) stationary phases. The panel on the left represents a biological replicate, and the results are consistent across all 3 replicates, as demonstrated in the panel on the right. Statistical significance observed between control and mutant groups (*P < 0.05, **P < 0.01, ***P < 0.001, 2-tailed t-test). **(b)** Redox activities of *E. coli* K-12 MG1655 WT, Δ*crp*, and Δ*cyaA* cells were measured at early (t=5h) and late (t=24h) stationary phases by flow cytometry using a RSG dye. This dye fluoresces green after reduction by bacterial reductases. A representative biological replicate is shown (left), with consistent results across all 5 replicates (right). Statistical significance observed between control and mutant groups (*P < 0.05, **P < 0.01, 2-tailed t-test). **(c)** *E. coli* cells with integrated mCherry expression system used to validate cellular respiration. Cells were diluted into fresh media and treated with ampicillin (200 μg/mL) for 20 hours. Flow cytometry measured the red fluorescence of intact surviving cells. A representative biological replicate is shown, with consistent results across all 3 replicates. **(d)** RSG levels of cells (carrying the mCherry expression system) at exponential phase (t=3h); cells before ampicillin treatment; non-lysed (intact) cells after 20 hours of ampicillin treatment; and untreated cells after 20 hours of culturing. A representative biological replicate is shown (left), with consistent results across all 4 replicates (right). Statistical significance observed between intact antibiotic-treated cells and others (*P < 0.05, **P < 0.01, 2-tailed t-test). **(e)** High throughput screening of mutants from Keio collection. The mutant strains selected are associated with central metabolism. Stationary phase cells were diluted 100-fold in fresh medium and treated with ampicillin (200 μg/mL) or ofloxacin (5 μg/mL) for 20 hours. Treated cultures were washed, serially diluted, and plated on agar plates to quantify CFUs. **(f)** Genes related to the TCA cycle, ETC, ATP synthase, glycolysis, and pentose phosphate pathway (PPP) were knocked out and then treated with ampicillin (200 μg/mL) or ofloxacin (5 μg/mL) to enumerate CFUs. n=4. Biphasic kill curves were generated using a non-linear model. Statistical significance tests were conducted using F-statistics (*P < 0.05, and **P < 0.01). Each data point represents the mean value ± standard deviation.

The Crp/cAMP-mediated metabolic state, characterized by increased respiration in WT compared to mutant strains, was further validated using redox sensor green (RSG) dye and a reporter plasmid measuring the promoter activity of succinate:quinone oxidoreductase (SQR) genes. The SQR reporter system ^52^ employs green fluorescent protein (GFP) expression, regulated by the promoter of the SQR operon, which includes the *sdhA, sdhB, sdhC,* and *sdhD* subunit genes. The SQR complex plays a vital role in cellular metabolism by catalyzing the oxidation of succinate to fumarate concurrently with the reduction of ubiquinone to ubiquinol, thus directly linking the TCA cycle with the respiratory ETC ^40^. Our results indicate an upregulation of the SQR promoter activity in WT cells compared to the mutant strains in the stationary phase, validating the findings from our metabolomics and proteomics data (Fig. 5a). The RSG dye, on the other hand, serves as a well-established metabolic indicator, measuring bacterial oxidation-reduction activity, a crucial function involving the ETC driven by the TCA cycle. Once reduced by bacterial reductases, the RSG dye emits a stable green fluorescent signal **(Supplementary Fig. 11).** Our data demonstrate that the redox activities of WT cells are much higher and more heterogeneous compared to those of the mutant strains in the late stationary phase, further corroborating the results from our preceding analyses (Fig. 5b). Furthermore, we utilized a methodology that integrates the mCherry expression system, flow cytometry, and ampicillin-mediated cell lysis, to determine whether persister cells in WT still maintain their respiration. In this assay, both the WT and mutant strains carrying the mCherry expression system were exposed to ampicillin after transferring them to a fresh medium. The inducer was added to both growth and treatment cultures to sustain the cells' red signals. Unlike other antibiotics, ampicillin disrupts cell wall synthesis, leading to the lysis of cells upon their resumption of growth. As seen in **Supplementary Fig. 12,** the cells that were lysed lost their mCherry signals. On the other hand, the resilient, tolerant cells that evaded ampicillin-induced lysis maintained their mCherry levels throughout the treatment. In the mutant strains, ampicillin was effective in lysing almost all cells as anticipated, leaving no or small number of intact cells **(Supplementary Fig. 12).** However, in the WT strain, we detected a subpopulation of intact cells throughout the entire treatment period (Fig. 5c, and **Supplementary Fig. 12**). While the population-level redox activities of these tolerant intact cells in WT are lower than those of exponential phase cells, they still displayed a significant increase in RSG levels compared to cell populations before antibiotic treatments or untreated control cells subjected to identical conditions (Fig. 5d), suggesting that they maintain steady-state energy metabolism. We want to highlight that not all intact cells in the WT strain reported here are persisters. A significant portion comprises 'viable but non-culturable' (VBNC) cells, and WT cells exhibit markedly higher VBNC levels than Δ*crp* and Δ*cyaA* mutant strains **(Supplementary Fig. 13).** VBNC cells can be quantified from intact cells following beta-lactam treatments ^30,50^. These cells may exhibit metabolic activities but are unable to readily colonize upon transfer to fresh medium ^30,53^. Collectively, our metabolic measurement data (Fig. 5a, b, c) aligns with the findings from our omics analyses, and the number of intact cells observed in WT after beta-lactam treatment is consistent with the count of non-growing cells in WT (Fig. 4b vs *Supplementary Fig. 12*). These non-growing cells are anticipated to be less susceptible to lysis by beta-lactams ^30,50^.

### The genomic-level screening of *E. coli* knockout underscores the significance of energy metabolism in sustaining the viability of persister cells.

Although some metabolic genes, including those encoding the TCA cycle (e.g., *sdhA, sucB, mdh, icd*), have been studied in *E. coli* ^9,10,23,54,55^, a comprehensive genomic-level screening strategy is necessary to validate which metabolic pathways are truly associated with antibiotic tolerance. To further underscore the importance of energy metabolism, we conducted a high throughput screening of 149 different *E. coli* K-12 BW25113 mutant strains from the Keio knockout library ^56^. The selected strains are related to central carbon metabolism, encompassing glycolysis, pentose phosphate pathways, TCA cycle, ETC, ATP synthase, and fermentation pathways (Fig. 5e). While the deletion of genes related to glycolysis and pentose phosphate pathways did not affect antibiotic tolerance in the cells, some mutant strains associated with cytochrome bo and quinone oxidoreductase complexes (e.g., *cyo* genes, and *nuoL*) exhibited enhanced tolerance **(Supplementary Table 6).** However, the mutant strains exhibited reduced tolerance to both antibiotics compared to the control K-12 BW25113 WT strain were found to be largely associated with the TCA pathway (*sucA, sucB, lpd, sucC, sucD, sdhA, sdhB, sdhC, sdhD, gltA, acnB, aceE, fumA, mdh* and *fumC*), ETC (*nuoB, nuoC, nuoI, nuoK, nuoM,* and *narV*), ATP synthesis (*atpA, atpB, atpC, atpD, atpE,* and *atpH*), and mixed acid fermentation pathways (*ldhA, fdhF, pta, adhE,* and *frdC*) (Fig. 5e, and **Supplementary Table 6**). We acknowledge that the Keio strains were generated in a high-throughput manner, and there might be unknown errors in their genomic DNA. To ensure the reproducibility of our findings, we generated knockout strains for key genes associated with the TCA, ETC, ATP synthase, glycolysis, and pentose phosphate pathway (Fig. 5f, see **Supplementary Table 7** for detailed description of genes). We then tested their antibiotic tolerance, and our results were consistent with the omics data and screening outcomes (Fig. 5f), confirming the critical role of energy metabolism, specifically the TCA cycle, ETC, and ATP synthase, in bacterial persistence.

## DISCUSSION

Our study highlights the crucial role of the Crp/cAMP complex in maintaining the metabolic state of stationary-phase persister cells, enabling their survival under adverse conditions. Through metabolomics, proteomics, and high-throughput screening of single-gene deletion strains, we substantiated that the Crp/cAMP regulatory complex sustains an active respiratory state while downregulating anabolic pathways in persister cells. This respiratory state is vital for the survival of persister cells, as perturbing the Crp/cAMP complex or respiration significantly reduced persister phenotypes, which may explain the previously reported decrease in antibiotic tolerance in *E. coli* cells cultured under anaerobic conditions ^10^. Notably, we observed an upregulation of anabolic metabolites and proteins when the Crp/cAMP regulatory complex was perturbed, particularly those associated with cell wall organization, cell cycle, and cell division, enhancing the ability of stationary-phase mutant cells to resume growth. Although the literature has shown associations between antibiotic tolerance with proteins involved in cell division and the TCA cycle (e.g., SdhA, SucB, Mdh) ^9,10,54,55^, our study establishes a strong link between these critical cellular processes and the Crp/cAMP complex, providing much-needed clarity in the field. In fact, upon investigating Crp/cAMP regulons via the Ecocyc database ^40^, we identified that certain metabolic genes, deleted in our *E. coli* K-12 MG1655 background, are potentially regulated by Crp/cAMP **(Supplementary Table 7),** providing additional support for the validity of our omics results. While our discoveries enhance the comprehension of the Crp/cAMP-regulated metabolic network and its implications for antibiotic tolerance, it is essential to address several noteworthy highlights:

**(i)** The deletion of *cyaA* resulted in a reduction of cAMP levels, as expected given the role of the CyaA enzyme in cAMP synthesis **(Supplementary Fig. 1).** Conversely, the removal of *crp* led to an increase in cAMP levels compared to those in wild-type cells. Notably, this increase is statistically significant **(Supplementary Fig. 1),** and to the best of our knowledge, this phenomenon has not been described before. We propose two potential reasons for this observation. Firstly, the *cyaA* promoter is recognized to be inhibited by the Crp/cAMP complex ^39,40^. The absence of Crp may compromise the formation of the Crp/cAMP complex, potentially enhancing the expression of the CyaA enzyme and consequently increasing cAMP concentration. The second possibility involves a potential inhibition of the cAMP degradation pathway in the Δ*crp* mutant. It is noteworthy that the enzyme responsible for cAMP hydrolysis is cAMP phosphodiesterase, and whether its corresponding gene, *cpdA*, is regulated by the Crp/cAMP complex remains unknown.

**(ii)** Our findings reveal substantial heterogeneity in metabolism (measured by RSG) among WT stationary phase cells, contrasting with the more uniform behavior observed in Δ*crp* and Δ*cyaA* strains (Fig 5b). We also demonstrated the presence of two distinct populations in WT cells during the late stationary phase: one that resumes rapid growth and another subpopulation that does not resume growth when transferred to a fresh medium (Fig. 4b). The absence of this non-growing cell subpopulation in the mutant strains could account for their sensitivity to aminoglycosides. However, the mechanism by which aminoglycosides kill these non-growing cells in the WT strain remains perplexing. The underlying reasons might be linked to their metabolism, as aminoglycoside uptake is an energy-requiring process, relying on the electron flow through membrane-bound respiratory chains ^29^. Moreover, persister cells obtained from various antibiotics, such as ampicillin and ofloxacin, in WT *E. coli* were previously found to exhibit sensitivity to aminoglycosides when sugar molecules were introduced into the cultures ^29,30^. However, the enhanced sensitivity mediated by sugar molecules was reversed to its original state in a subsequent study when the Crp/cAMP complex was genetically perturbed ^31^. This can be attributed to the lack of active energy metabolism in these genetically altered strains, as suggested by our comprehensive genomic-level analyses here. While the absence of cell division in non-growing cell subpopulations in WT may suggest a downregulation in anabolic metabolism, their energy metabolism may remain partially active, which could potentially explain the phenomenon of aminoglycoside potentiation. Indeed, our results presented in Fig. 5d support this interpretation.

**(iii)** Antibiotics are generally effective against proliferating bacteria, leading to the notions that tolerance is linked to temporary growth suppression and that persister cells are dormant phenotypes with repressed metabolism. Our previous studies utilizing a redox sensor and fluorescent-activated cell sorting revealed that, while persister cells were predominantly enriched in cell subpopulations with high redox activities in stationary phase cultures (consistent with our findings) ^10^, the opposite was observed in exponential phase cultures ^11^. Indeed, this aligns with the flow diagram presented in Fig. 5d in the current study, wherein persister cells exhibit a lower metabolic rate compared to rapidly growing exponential-phase cells and a higher metabolic rate compared to non-growing stationary-phase cells. While several independent studies have demonstrated that persister cells exhibit reduced metabolic activities compared to exponentially growing cells ^23,57^, the direct comparison of persister cell metabolism to that of exponentially growing cells may not be the best approach. Growing cells have a very high energy output and consume metabolites at a fast pace. Therefore, any comparison between tolerant and non-tolerant cell populations requires proper normalization techniques such as adjusting cellular metabolic activities to the amount of substrate utilized by cells. An example of this normalization was conducted by Heinemann's group ^58^, demonstrating that ATP production rates per substrate in tolerant cells exceed those of exponentially growing cells.

**(iv)** We diluted cells in fresh medium at early and late stationary phases before antibiotic treatments. This step is essential for quantifying type I persisters, as these cells do not readily resume growth upon dilution in the fresh medium during the lag phase ^1,10^. We acknowledge that antibiotic tolerance is influenced by various factors, including culture dilutions, media, specific strains, antibiotics tested, treatment durations, and the growth phase during treatment administration. These factors may contribute to variations in reported persister levels observed in the Δ*cyaA* strain during the exponential phase ^59-62^. While we did not focus on the exponential growth phase in our study, it is noteworthy that the reduced growth rate during this phase in the mutant strains (Fig. 4c) may explain the antibiotic tolerance observed in previous studies involving the *cyaA* deletion ^59-62^. The growth disparity noted between mutant and WT strains, particularly evident around 5 hours in our results **(Supplementary Fig. 2),** may also be linked to the ofloxacin persisters observed in the mutant strains at this specific time point (Fig. 1). While slow cell growth may indeed correlate with bacterial persistence ^63^, it is important to note that the persistence associated with perturbations of metabolic genes cannot be solely attributed to the slow growth. In fact, the persistence of these mutant strains should depend on many factors (see **Supplementary Table 7)** as reported by diverse research groups ^6,9,10,54,55,64-68^. For instance, in *E. coli*, TCA inactivation was shown to decrease ampicillin and ofloxacin persistence during the lag phase ^10^, yet it enhances gentamicin tolerance in the exponential phase, which remains unexplained by factors such as cell growth, redox activities, proton motive force (PMF), or ATP levels ^69^. Furthermore, gene deletions often trigger pleiotropic effects, leading to unique tolerance mechanisms not evident in wild-type strains. In our Keio screening data analysis, we observed that the deletion of *icd* appeared to enhance persistence **(Supplementary Table 6),** in line with a previous study ^23^. The *icd* gene encodes a TCA cycle enzyme, isocitrate dehydrogenase. While it remains unclear whether this observed outcome is attributable to other unseen pleiotropic effects stemming from the *icd* deletion, our data consistently indicates that the most significant reduction in persistence levels occurs with disruptions in energy metabolism. A comprehensive approach, encompassing omics and knockout screening as presented in this study, offers a more complete understanding, revealing the consensus behavior within the entire metabolic network.

In conclusion, a significant gap in the current literature is the lack of a comprehensive understanding of how bacterial cell metabolism undergoes changes during the transition to a tolerant state. Identifying the specific metabolic pathways that gain significance for cell survival in this context is crucial. This knowledge can pave the way for the development of more informed and targeted treatment strategies, ultimately enhancing our ability to combat tolerant cells and improve overall treatment outcomes.

## MATERIALS AND METHODS

### Bacterial Strains and Plasmids

All experiments were conducted using *E. coli* K-12 MG1655 wild-type (WT) and its derivative strains. *E. coli* K-12 MG1655, MO strains (carrying the mCherry expression system) and pUA66 plasmids were obtained from Mark P. Brynildsen at Princeton University. *E. coli* MO strain was used to monitor cell proliferation at single cell level due to its chromosomally integrated isopropyl β-D-1-thiogalactopyranoside (IPTG)-inducible mCherry expression cassette ^10,11,30^. *E. coli* K-12 BW25113 WT and single deletions were obtained from Dharmacon Keio Collection (Dharmacon, Catalog# OEC4988, Lafayette, CO, USA). The mutant strains in this study were generated using the Datsenko-Wanner method ^70^. The pUA66-EV was generated by the removal of *gfp* gene from the plasmid. The *crp* gene with its promoter was cloned into the modified pUA66 plasmid to obtain the pUA66-*crp* expression system. The *SdhABCD* reporter (pMSs201-P_*sdhABCD*_-*gfp*) was obtained from a previous study ^52^. The cloning method was followed according to a standard method from NEB ^71^. Genetic modifications were verified by PCR and gene sequencing (Genewiz, South Plainfield, NJ, USA). A complete list of strains, plasmids, and oligonucleotides used in this study is presented in **Supplementary Tables 8** and **9.**

### Media, Chemicals, and Culture Conditions

All chemicals used in this study were purchased from Fisher Scientific (Atlanta, GA, USA), VWR International (Pittsburg, PA, USA), or Sigma Aldrich (St. Louis, MO, USA). Luria-Bertani (LB) medium was prepared by combining 5 g of yeast extract, 10 g of tryptone, and 10 g of sodium chloride in 1 L of autoclaved deionized (DI) water. LB agar media was prepared by mixing 40 g of pre-mixed LB agar with 1 L of autoclaved DI water; LB aga media were used to enumerate colony-forming units (CFUs) ^10,41,72^. For washing cells and removing chemicals and antibiotics before plating on agar media, 1X Phosphate-Buffered Saline (PBS) was employed. In the persister assay, concentrations of 5 μg/mL of ofloxacin (OFX), 200 μg/mL of ampicillin (AMP), and 50 μg/mL of gentamicin (GEN) were used ^29,41,73,74^. The retention of plasmids necessitated 50 μg/mL of kanamycin (KAN) in the culture media ^10^. Fluorescent protein expression was induced using 1 mM ITPG ^10^. Overnight pre-cultures were prepared in 14 mL Falcon test tubes containing 2 mL of LB medium, inoculated from a 25% glycerol cell stock stored at −80°C, and incubated for 24 hours at 37°C with shaking at 250 revolutions per minute (rpm). Main cultures were established by diluting the overnight pre-cultures at a ratio of 1:1000 into 2 mL fresh LB medium in 14 mL Falcon test tubes. Experimental cell cultures were prepared by further dilution of the main cultures into either 25 mL fresh LB medium in 250 mL baffled flasks or 2 mL fresh LB medium in 14 mL Falcon test tubes. Cultures at t=5h and t=24h were defined as early and late stationary phase cultures, respectively. Detailed experimental procedures are outlined below.

### Cell growth and persister assays

Main cultures were prepared by diluting the overnight pre-cultures at a ratio of 1:1000 into 2 mL of fresh LB medium in 14 mL Falcon test tubes. These cultures were then incubated at 37°C with shaking at 250 rpm. Cell growth was monitored by measuring the optical density at 600 nm wavelength (OD_600_) using a Varioskan LUX Multimode Microplate Reader (Thermo Fisher, Waltham, MA, USA). The plate reader data was collected using Skanlt Software V 5.0. Cell cultures at both early and late stationary phases were collected from the test tubes and transferred to the baffled flasks to achieve ~5x10^7^ cells/mL. This concentration represents an approximately 100-fold dilution of the WT main culture into a fresh medium within the flask. It is important to highlight that we consistently employed flow cytometry to quantify the initial cell count (refer to the section "Monitoring cell division" for comprehensive details). As needed, adjustments in cell number-to-volume were executed to ensure the same cell number among both WT and mutant strains. These cultures were then treated with antibiotics at the indicated concentrations and were cultured with shaking at 37°C for 20 hours. After the treatment, a 1 mL sample from each flask was transferred to a microcentrifuge tube and centrifuged at 13,300 rpm (17,000 x gravitational force or g). The supernatant (900 μL) was removed, and the cells were washed twice with 1X PBS to eliminate antibiotics from the sample. Following the washes, 100 μL of 1X PBS was used to resuspend the cells. A 10 μL sample of this cell suspension was serially diluted in a 96-well round-bottom plate. Then, 10 μL from each dilution was spotted on an agar plate, and the remaining 90 μL from the most concentrated well was plated to ensure viable cell detection down to a limit equivalent to 1 CFU/mL. These plates were incubated for 16 hours at 37°C to allow CFUs to develop.

### cAMP profile assay

An overnight pre-culture of *E. coli* K-12 MG1655 WT was prepared in test tubes with 2 mL of LB medium. The culture was incubated at 37°C with shaking at 250 rpm for 24 hours. For the experimental cultures, a 1:1000 dilution was made in fresh medium. At time-points 0, 2, 4, 6, 8 and 24h, 100 μL of the experimental culture was washed with cold 1X PBS. After centrifuging at 13,300 rpm (17,000 x gravitational force or g), the supernatant was removed. The cells were then resuspended in 100 μL of cell lysis buffer from the Cyclic AMP XP^®^ Assay Kit (Catalog# 4339S, Cell Signaling Technology, Danvers, MA, USA) on ice for 10 minutes. Next, 50 μL of lysed cells was mixed with the kit’s horseradish peroxidase (HRP)-linked cAMP solution in the cAMP assay plate. This mixture was incubated at room temperature for 3 hours on a plate shaker at 250 rpm. Following the 3-hour incubation, the plate content was discarded, and the plate was washed four times with the kit’s Wash Buffer. Then, 100 μL of tetramethylbenzidine (TMB) substrate was added to allow color development, and after 30 minutes, 100 μL of the stop solution provided by the kit was added. The absorbance was measured at 450 nm using a plate reader. The standard curve was prepared using the same conditions and the standard cAMP solutions provided by the kit to determine cAMP concentrations.

### Metabolomics

Metabolites from both Δ*crp* and WT cells were analyzed at the Metabolon, Inc., facility (Morrisville, NC, USA). Cells were cultured until the early stationary phase and late stationary phase at 37°C with shaking at 250 rpm. Afterward, cells were collected through centrifugation at 4700 rpm at 37°C for 15 minutes, yielding a pellet of approximately 100 μL containing around 10^10^ cells. Subsequently, the cells were washed once with 1X PBS and then centrifuged (13,000 rpm, 3 minutes at 4°C). Following this, they were frozen in an ethanol/dry ice bath for 10 minutes. The extracts from both mutant and WT cells were subjected to analysis using ultra-high-performance liquid chromatography-tandem accurate mass spectrometry (MS), a process aimed at identifying a wide range of metabolites. Sample extraction, preparation, instrument settings, and conditions for the MS platform adhered to Metabolon’s protocols (as detailed in our previous study) ^75^. To identify the sample’s metabolites among potential false positives from instrument noise, process artifacts, and redundant ion features, the results were cross-referenced with Metabolon’s extensive metabolite library (standards). Data normalization was carried out based on protein concentration, determined using the Bradford assay. The significant difference between mutant and WT was identified using Welch’s two-sample t-test. Further analysis involved pathway enrichment assessment using MetaboAnalyst ^47^. This involved inputting the upregulated and downregulated metabolites based on chosen thresholds. A comprehensive overview of metabolite measurements, pathway enrichment, statistical analyses, and data representations can be found in our previously published study ^75^.

### Proteomics

Overnight cultures for both WT and Δ*crp* were prepared using 2 mL of LB medium. Incubation was carried out at 37°C and 250 rpm for 24 hours. The following day, the main cultures were established under the same conditions, using a 1000-fold dilution of the overnight culture in 2 mL fresh LB medium. After 24 hours, the OD_600_ of both WT and mutant strains was measured and adjusted to an OD_600_ of 2.5. For further processing, 2 mL of the main culture was washed twice with cold 1X PBS, maintaining the cold environment throughout. Centrifugation conditions were set at 4°C, 13,000 rpm for 3 minutes. Before the final centrifugation, a cell count was conducted using flow cytometry. This involved using 10 μL of washed culture and 990 μL of 1X PBS. Subsequent to centrifugation, the pellets were collected. Cell lysis was carried out using 300 μL of NEBExpress^®^ *E. coli* Lysis Reagent (Catalog# P8116S, Ipswich, MA, USA) at room temperature for 30 minutes. Following this, the lysed samples were centrifuged at 16,600 x g for 10 minutes, and 250 μL of supernatants were collected from each sample for the assay. The total protein concentration of the supernatants was determined using the bicinchoninic acid (BCA) assay (Catalog# 23225, Thermo Fisher Scientific, Waltham, MA, USA). In a 96-well plate, 25 μL of each cell lysate sample, diluted 5 and 10 times with ultra-pure DI water, were loaded into each well. Subsequently, 200 μL of the BCA working reagent (50:1, Reagent A:B) was added to each well and mixed on a plate shaker for 30 seconds. The plate was then incubated at 37°C for 30 minutes, followed by cooling for 5 minutes at room temperature, shielded from light. The absorbance was finally measured at 562 nm using a plate reader, and the total protein concentration in each sample was calculated using a standard curve prepared from standard protein solutions. Protein analysis for both WT and mutant was conducted by the proteomics service at UT Health's Clinical and Translational Proteomics Service Center (Houston, TX). The samples underwent acetone precipitation, during which proteins were precipitated by exposing them to −20°C for 3 hours. Following this, a centrifugation step at 12,000 g for 5 minutes separated the precipitated pellets. These pellets were subsequently subjected to denaturation and reduction using a mixture containing 30 μL of 6 M urea, 20 mM DTT in 150 mM Tris HCl (pH 8.0) at 37°C for 40 minutes. Afterward, alkylation was carried out with 40 mM iodoacetamide in the absence of light for 30 minutes. To prepare for digestion, the reaction mixture was diluted 10-fold using 50 mM Tris-HCl (pH 8.0) and then incubated overnight at 37°C with trypsin at a 1:30 enzyme-to-substrate ratio. The digestion process was terminated by adding an equal volume of 2% formic acid, followed by desalting using Waters Oasis HLB 1 mL reverse phase cartridges, following the vendor's recommended procedure. Finally, the eluates were dried using vacuum centrifugation. Approximately 1 μg of the tryptic digest, prepared in a solution containing 2% acetonitrile and 0.1% formic acid in water, underwent analysis using LC/MS/MS. The instrument used was the Orbitrap Fusion^™^ Tribrid^™^ mass spectrometer by Thermo Scientific^™^, connected to a Dionex UltiMate 3000 Binary RSLCnano System. The separation of peptides occurred on an analytical C18 column with dimensions of 100 μm ID x 25 cm, featuring 5 μm particles and an 18 Å pore size. Peptides were eluted at a flow rate of 350 nL/min. The gradient conditions applied were as follows: a gradient starting from 3% B and increasing to 22% B over a duration of 90 minutes, followed by a step to 22%-35% B for 10 minutes, then another step to 35%-90% B for 10 minutes, and finally, maintaining 90% B for an additional 10 minutes (Solvent A was composed of 0.1% formic acid in water, while solvent B contained 0.1% formic acid in acetonitrile). The peptides were analyzed using a data-dependent acquisition method. The Orbitrap Fusion MS operated by measuring FTMS1 spectra with a resolution of 120,000 FWHM, scanning in the m/z range of 350-1500, using an AGC target set to 2E5, and with a maximum injection time of 50 ms. Within a maximum cycle time of 3 sec, ITMS2 spectra were collected in rapid scan mode. High Collision Dissociation (HCD) was employed with a normalized collision energy (NCE) of 34, an isolation window of 1.6 m/z, an AGC target set to 1E4, and a maximum injection time of 35 ms. Dynamic exclusion was implemented for a duration of 35 sec to prevent repeated analysis of the same ions. For the experimental analysis, the Thermo Scientific^™^ Proteome Discoverer^™^ software version 1.4 was utilized to process the raw data files. The spectra were subjected to analysis against the *E. coli* proteome database (Swiss-Prot 29,161) through the Sequest HT search engine. Additionally, the spectra were compared against a decoy database, employing a target false discovery rate (FDR) of 1% for stringent criteria and 5% for more relaxed criteria. The enzymatic cleavage allowance for trypsin included up to two potential missed cleavages. The MS tolerance was defined as 10 ppm, while the MS/MS tolerance was set at 0.6 Da. Fixed modification involved carbamidomethylation on cysteine residues, and variable modifications encompassed methionine oxidation and asparagine deamidation. For proteomics data processing and fold change calculations, the approaches were essentially followed by the method paper from Aguilan *et al* ^76^ . Then, the STRING tool V 12.0 ^48,49^ was employed to find the significant networks among input proteins. To generate the protein network and pathway enrichment analysis, we input the protein identifiers (Accession numbers) for upregulated or downregulated proteins with at least a 2-fold increase or reduction, respectively. *E. coli* K-12 was selected as the organism of interest. We opted for evidence as the criterion for network edges, prioritizing the type of interaction evidence, helping us conduct an automated pathway-enrichment analysis, centering on the entered proteins and identifying pathways that occurred more frequently than expected. This analysis was grounded in the statistical background of the entire genome and encompasses various functional pathway classification frameworks, such as Gene Ontology annotations, KEGG pathways, and Uniprot keywords, as detailed elsewhere ^48,49^. In pathway enrichment analysis, the "strength score," calculated as Log10(observed/expected), serves to assess the degree or significance of enrichment within a specific biological pathway. This metric reflects the magnitude of the enrichment effect, with a higher score indicating stronger enrichment. The score is derived from the ratio of i) annotated proteins in the network for a given term to ii) the expected number of proteins annotated with the same term in a random network of equivalent size ^48,49^. To gauge the significance of enrichment, False Discovery Rate (FDR) is employed. FDR scores represent p-values corrected for multiple testing within each category using the Benjamini–Hochberg procedure ^48,49^.

### Monitoring cell division

To monitor cell division and quantify non-growing cells, we utilized inducible fluorescent protein (mCherry) expression. Overnight pre-cultures of *E. coli* MO were prepared with 2 mL of LB medium containing 1 mM of ITPG. These cultures were grown in test tubes at 37°C with shaking at 250 rpm for 24 hours. Main cultures were established by diluting the overnight pre-cultures (at a ratio of 1:1000) into 2 mL of fresh LB medium in 14 mL Falcon test tubes. These cultures were incubated at 37°C with shaking at 250 rpm. Cells were allowed to grow until they reached the early stationary phase and the late stationary phase. The mCherry positive cells were then collected, washed twice with 1X PBS to remove the ITPG from the culture, and subsequently resuspended in fresh 2 ml LB media in test tubes to achieve ~5x10^7^ cells/mL. This concentration represents an approximately 100-fold dilution. When needed, adjustments in cell number-to-volume were made to ensure the same cell number among both WT and mutant strains. In the experimental culture test tubes, 2 mL of LB medium were added, and the volume of washed cells was inoculated to achieve an OD_600_ of 0.0286. The culture was then incubated at 37°C with shaking at 250 rpm. At specific time points (0, 1, 2, and 2.5h), cells were collected and resuspended in 1X PBS to measure their fluorescent protein content using flow cytometry. For flow cytometry analysis, cells were collected and diluted to a desired cell density (~10^6^-10^7^ cells/mL) in 1 mL of 1X PBS in flow cytometry tubes (5 mL round-bottom Falcon tubes, size: 12 x 75 mm). The flow cytometry analysis was conducted using a NovoCyte 3000RYB instrument (ACEA Bioscience Inc., San Diego, CA, USA). During flow cytometry analysis, a slow sample flow rate of 14 μL/min was chosen, along with a sample stream diameter (core diameter) of 7.7 μm. The instrument maintained a constant sheath flow rate of 6.5 mL/min. The core diameter was calculated using the ratio of the sample flow rate to the sheath flow rate. These specific conditions were selected to achieve improved data resolution for the size of *E. coli* cells. Flow diagrams utilized forward and side scatter signals from viable cells, alongside a control of solvent devoid of cells, to ascertain the presence of cells. For the flow cytometry analysis, cells were excited at a 561 nm wavelength, and the red fluorescence was detected using a 615/20 nm bandpass filter.

### Cell growth measured by flow cytometry

Overnight cultures of *E. coli* K-12 MG1655 MO WT and MO mutant strains were diluted at a ratio of 1:1000 into 2 mL of fresh LB medium, placed in 14 mL Falcon test tubes, and incubated at 37°C with shaking at 250 rpm for 24 hours. For the main cultures, a similar strategy was employed. The cultures were diluted at a ratio of 1:100 into 2 mL of fresh LB medium in 14 mL Falcon test tubes. These cultures were then incubated at 37°C with shaking at 250 rpm. At specific time points, including t=0, 20 min, 40 min, and 1 to 5h, the cell growth was halted. This was achieved by diluting the cells in 1X PBS containing 25 μg/mL of chloramphenicol (CAM). The CAM treatment allowed for subsequent analysis without further division. Flow cytometry was then utilized to measure the number of cells at each of these time points. This approach provided insight into cell division dynamics and allowed for the quantification of cell populations under specific conditions.

### Fluorescent protein expression assay for reporter genes

Mutant and control strains were derived from *E. coli* K-12 MG1655 and carried pMSs201-*gfp* plasmids incorporating a P_*sdhABCD*_ gene promoter. Overnight pre-cultures were prepared using 2 mL of LB medium supplemented with 50 μg/mL KAN. These cultures were incubated in test tubes within a shaker at 37°C for 24 hours. Main cultures were established by diluting the overnight pre-cultures at a ratio of 1:1000 into 2 mL of fresh LB medium within 14 mL Falcon test tubes. These main cultures were maintained at 37°C with shaking at 250 rpm. Cell cultures at the desired growth phase were collected and then diluted to attain a desired cell density of around 10^6^-10^7^ cells/mL in 1mL of 1X PBS within flow cytometry tubes. This allowed for subsequent flow cytometry analysis, using the same conditions as described earlier for monitoring cell division (refer to “Monitoring cell division”). During analysis, a laser emitting light at 488 nm was used to excite the cells, and the resulting green fluorescence was detected using a 530/30 nm bandpass filter. This setup enabled the examination of the fluorescence patterns of the cells, offering insights into their dynamics under different conditions.

### Redox Sensor Green assay

To gauge bacterial metabolic activity, we employed the Redox Sensor Green (RSG) dye from Thermo Fisher (Catalog# B34954, Thermo Fisher Scientific, Waltham, MA). *E. coli* K-12 MG1655 WT and mutant cells from the desired growth phase were diluted at a ratio of 1:100 in 1 mL of 1X PBS. To this solution, 1 μL of the RSG dye was added to flow cytometry tubes. After a brief vortexing to ensure uniform mixing, the samples were incubated at 37°C in darkness for 10 minutes. Subsequently, these samples were subjected to flow cytometry analysis. For the flow cytometry analysis, the same methodology as employed in "Monitoring cell division" was followed, with one variation. Cells were excited at 488 nm during analysis, and the resulting green fluorescence was detected using a 530/30 nm bandpass filter. This setup allowed us to assess the fluorescence patterns, reflecting the metabolic activity of the bacterial cells under different conditions. As a control measure, cells were treated with 20 μM of carbonyl cyanide m-chlorophenyl hydrazone (CCCP) for 5 minutes before the addition of the RSG dye. This served to validate the assay's sensitivity to changes in metabolic activity **(Supplementary Fig. 11).**

### Metabolic activity of non-lysing cells and VBNC cell quantification

Overnight cultures of *E. coli* K-12 MG1655 MO WT and MO mutant strains were diluted at a ratio of 1:1000 into 2 mL of fresh LB medium supplemented with 1 mM ITPG. These cultures were established in 14 mL Falcon test tubes and incubated at 37°C with shaking at 250 rpm for 24 hours. Treatment cultures were prepared by diluting the main cultures at a ratio of 1:100 into 25 mL of fresh LB medium supplemented with 1 mM ITPG. These cultures were set up in 250 mL baffled flasks and contained 200 μg/mL AMP. They were then cultured at 37°C with shaking at 250 rpm for 20 hours. Both before and after the treatment, 1 mL samples were collected from the cultures. These samples were subjected to a washing procedure with 1X PBS to eliminate the antibiotic present in the samples. The washed cells were then resuspended in 1 mL of 1X PBS within flow cytometry tubes. To measure the metabolic activity of the non-lysing cells, the RSG dye was employed as described above. Intact cells (following antibiotic treatment), stained as live with RSG, comprised both persister and VBNC cells. Persister levels were quantified by plating the cells on an agar medium, as described previously ^30^. As VBNC cells cannot grow on agar medium, their enumeration involved subtracting the number of persister cells from the total number of intact cells.

### Screening *E. coli* (K-12 BW25113) Keio Knockout Collection

Overnight cultures of individual mutant strains, along with their parental strain K-12 BW25113 WT harboring a kanamycin-resistant marker, were diluted at a ratio of 1:1000 in fresh LB medium containing 50 μg/mL of KAN. This was done in 14 mL Falcon test tubes and the cultures were then incubated at 37°C with shaking at 250 rpm. Upon reaching the late stationary phase, cells were further diluted at a ratio of 1:100 in fresh medium supplemented with antibiotics at specified concentrations. These cultures were once again incubated at 37°C with shaking for 20 hours. Following the 20-hour treatment period, the same methodology described in the section "Cell growth and persister assays" was employed to quantify the number of persisters. This approach allowed for an assessment of the impact of antibiotics on the formation of persister cells for both mutant strains and the parental K-12 BW25113 WT strain.

### Persister quantitation in *E. coli* K-12 MG1655 single gene deletions

Overnight cultures of mutant strains were diluted at a ratio of 1:1000 in 14 mL Falcon test tubes containing 2 mL of LB medium. These cultures were then incubated at 37°C with shaking at 250 rpm. Upon reaching the late stationary phase, cells were diluted at a ratio of 1:100 in fresh medium supplemented with antibiotics at specified concentrations. The cultures were once again subjected to shaking at 37°C for 20 hours. The same method described earlier, referred to as "Cell growth and persister assays," was employed to quantify the number of persister cells resulting from this treatment. This approach allowed for the assessment of the impact of antibiotic exposure on persister cell formation within the mutant strains.

### Statistics and reproducibility

A nonlinear logarithmic model was employed to create biphasic kill curves ^18,77^. The significance of these kill curves was determined through the utilization of F statistics ^18,77^. Metabolomics data were subjected to analysis using Welch's two-sample t-test in order to identify metabolites that significantly differed between the control and mutant groups ^78^. For all experiments, a minimum of three independent biological replicates were conducted, unless explicitly stated otherwise. In each figure (excluding flow diagrams), the data for each time point are represented as the mean value accompanied by the standard deviation. In terms of statistical significance analysis, the designated threshold values for P were set as follows: *P < 0.05, **P < 0.01, ***P < 0.001, and ****P < 0.0001. All figures were generated using GraphPad Prism 10.3.0. The statistical analyses were carried out using the statistical functions of GraphPad Prism 10.3.0. For the clustering of metabolomics and proteomics data, the "Clustergram" function of MATLAB (V R2020b) was employed. FlowJo (V 10.8.1) was the tool used to analyze the data acquired from flow cytometry.

## Data Availability

All data in this manuscript can be found in either Main text or Supplementary file.
