# Supplementary figures and images for: UNRAVELING CRP/cAMP-MEDIATED METABOLIC REGULATION IN ESCHERICHIA COLI PERSISTER CELLS
Source: bioRxiv. 2025 Apr 8:2024.06.10.598332. Originally published 2024 Jun 10. Preprint. [Version 2] doi: 10.1101/2024.06.10.598332 (PMC11195080; doi:10.1101/2024.06.10.598332)

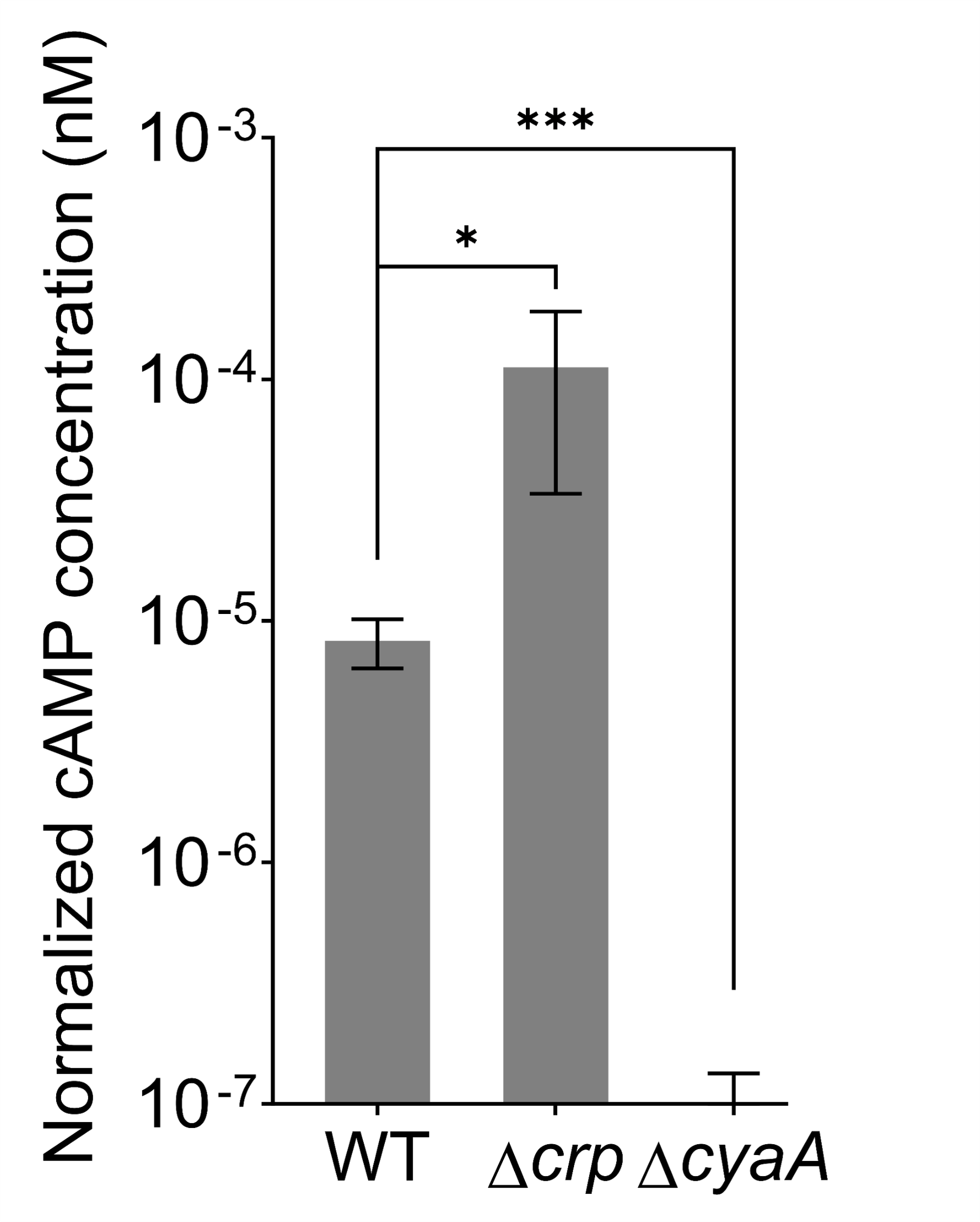

Supplement: Supplement 1 — Figure 1–figure supplement 1. cAMP concentrations normalized to cell numbers for E. coli K-12 MG1655 WT, Δcrp, and ΔcyaA. The cAMP levels were measured in late stationary phase cultures at 450 nm using the Cyclic AMP XP® Assay Kit (Cell Signaling Technology). n=4. Statistical significance was observed between control and mutant strains (*P < 0.05, ***P < 0.001, One-way ANOVA with Dunnett’s multiple comparisons test). The data for each time point represent the mean value ± standard deviation. [file media-1.tif]

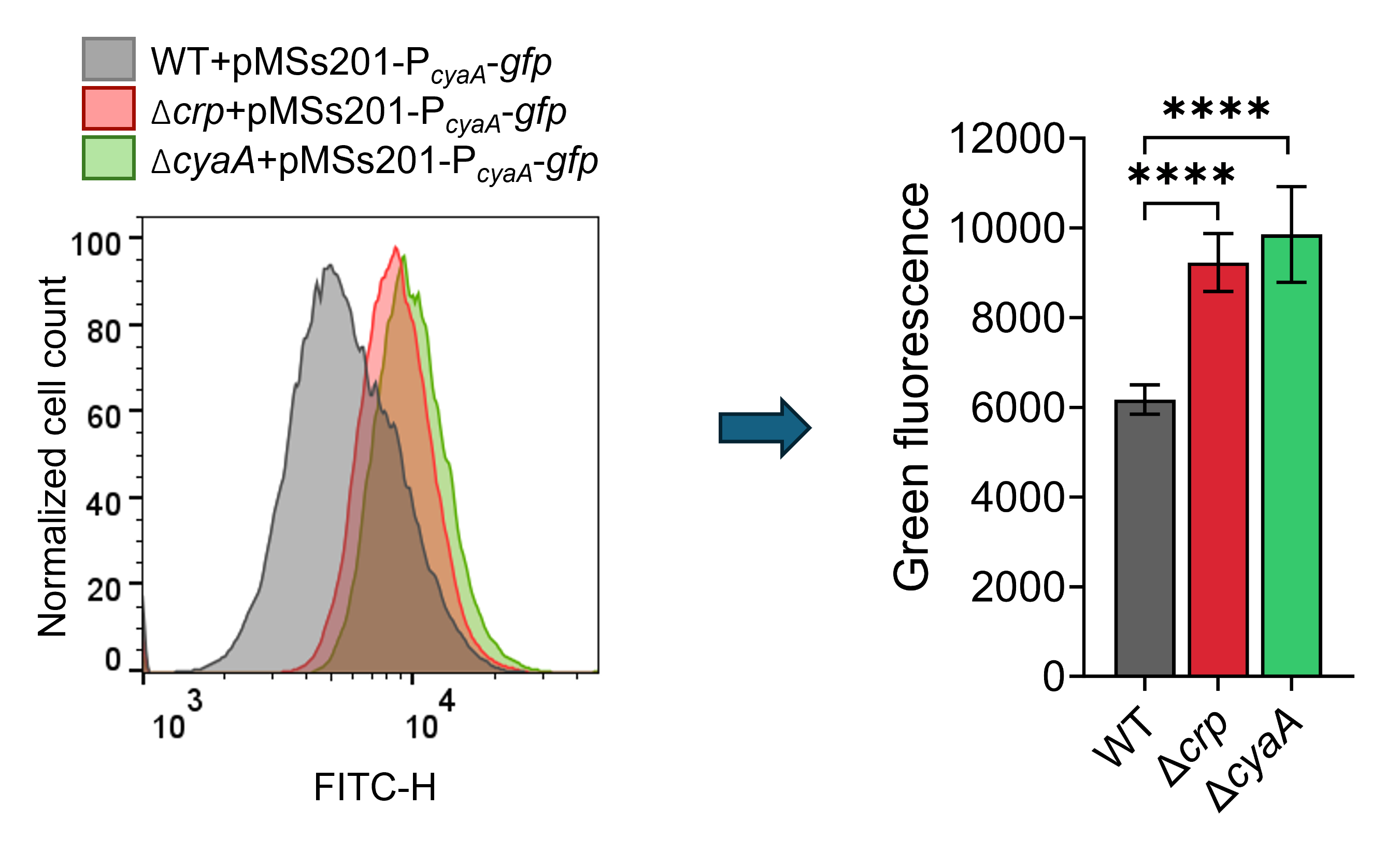

Supplement: Supplement 2 — Figure 1–figure supplement 2. Genetic perturbation of Crp/cAMP enhanced PcyaA promoter activity, resulting in increased gfp expression. Overnight cultures of E. coli K-12 MG1655 WT, Δcrp, and ΔcyaA strains harboring the pMSs201 plasmid, which encodes green fluorescent protein (GFP) under the control of the PcyaA promoter, were diluted 1:1000 into fresh LB medium and incubated at 37°C with shaking at 250 rpm for 24 hours. Cells at the late stationary phase were then collected, diluted in 1X PBS, and analyzed by flow cytometry. n=4. Statistical significance was observed between control and mutant strains (****P < 0.0001, One-way ANOVA with Dunnett’s multiple comparisons test). The data for each time point represent the mean value ± standard deviation. [file media-2.tif]

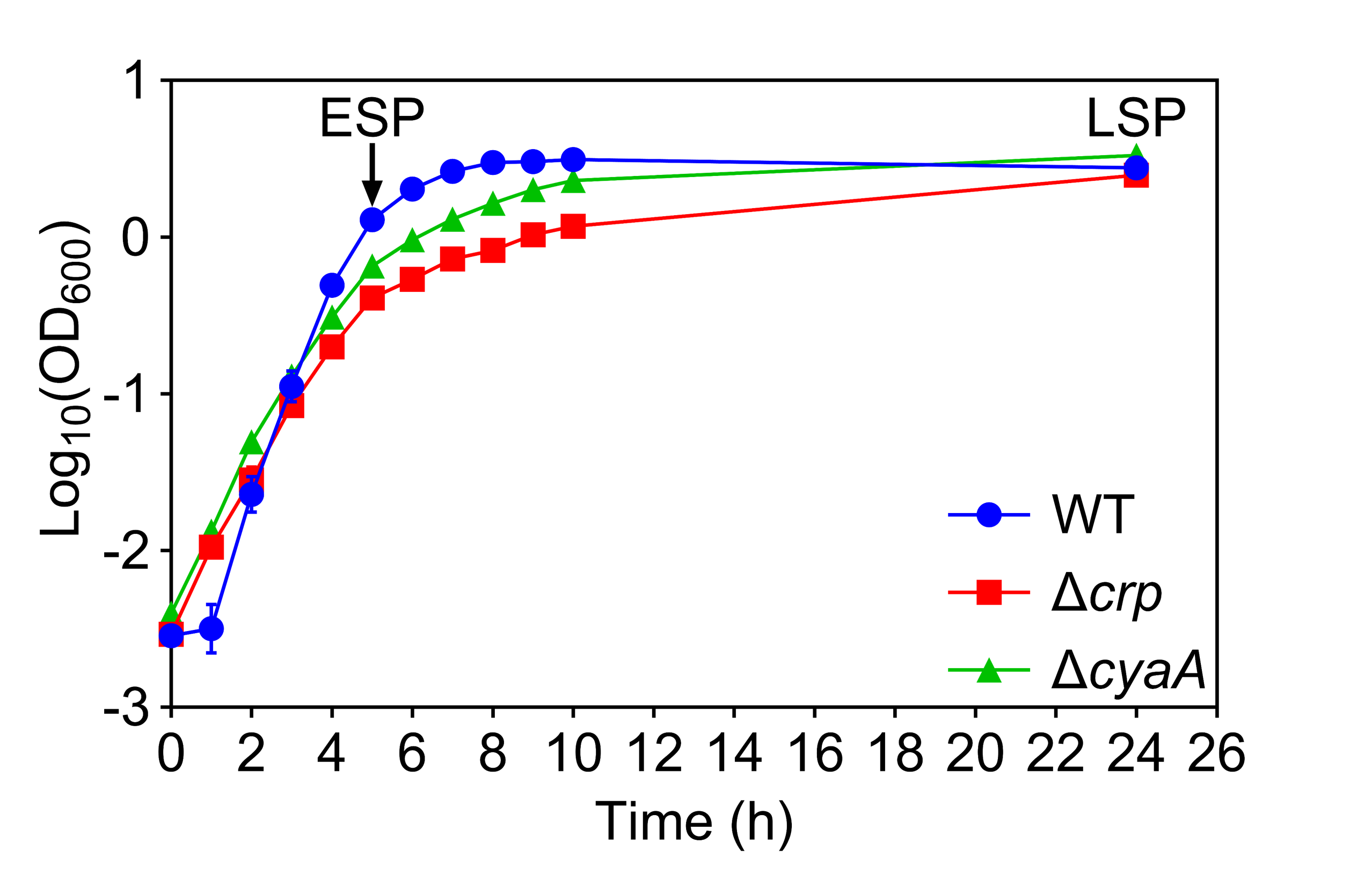

Supplement: Supplement 3 — Figure 1–figure supplement 3. Growth curves of E. coli K-12 MG1655 WT, Δcrp, and ΔcyaA. Optical densities of cell cultures at 600 nm (OD600) were measured every hour using a plate reader. n=3. The data for each time point represent the mean value ± standard deviation. [file media-3.tif]

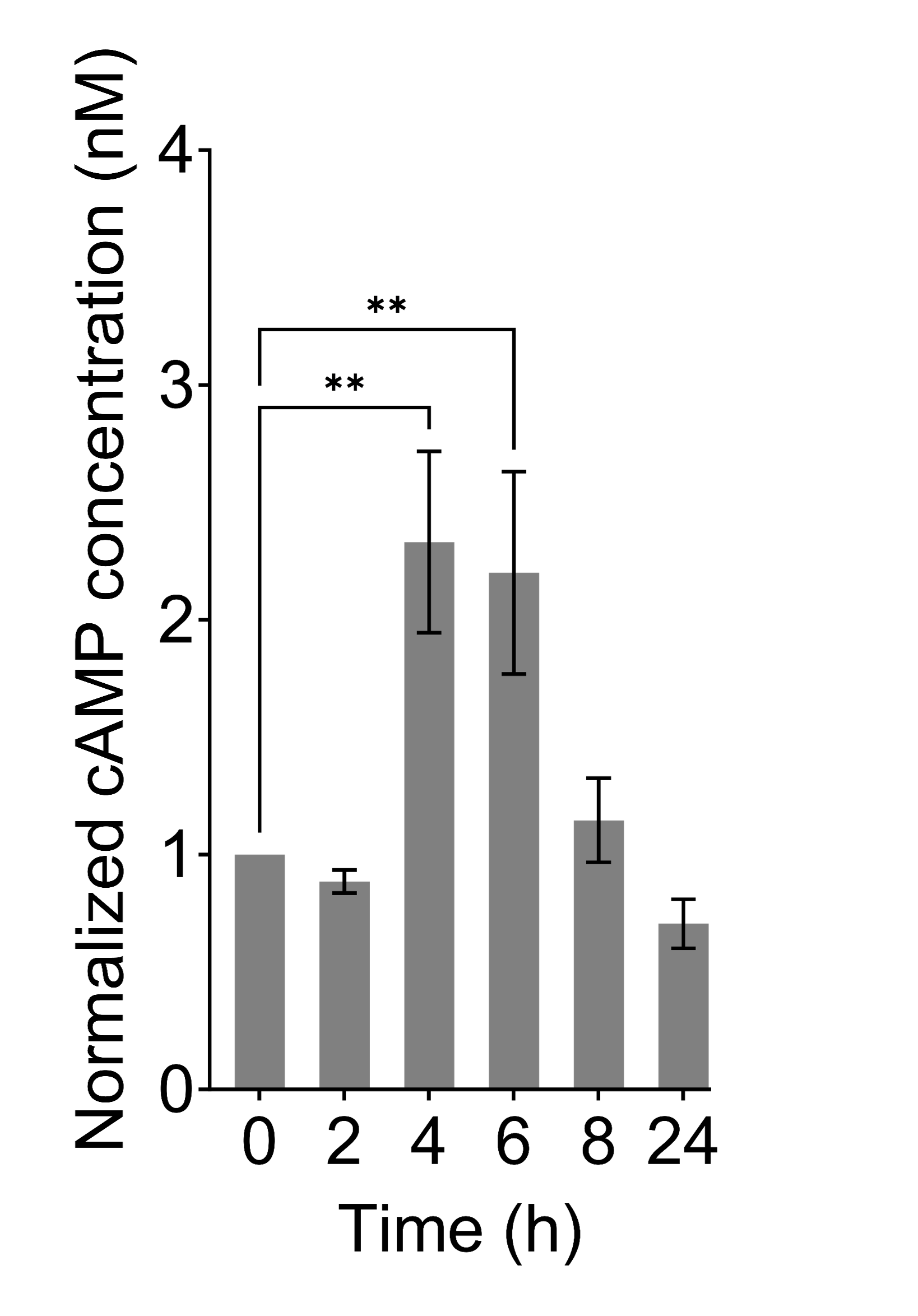

Supplement: Supplement 4 — Figure 1–figure supplement 4. Normalized cAMP concentrations of E. coli K-12 MG1655 WT. The cAMP concentrations were measured in growth cultures at the indicated time points. First, the cAMP concentrations were normalized to the number of cells. Subsequently, the data were further normalized based on the time point 0 to mitigate errors associated with batch-to-batch assay kit variations and to capture the trend in cAMP levels across the time points. n=8. Statistical significance was observed between time points (**P < 0.01, One-way ANOVA with Dunnett’s multiple comparisons test). The data for each time point represent the mean value ± standard error. [file media-4.tif]

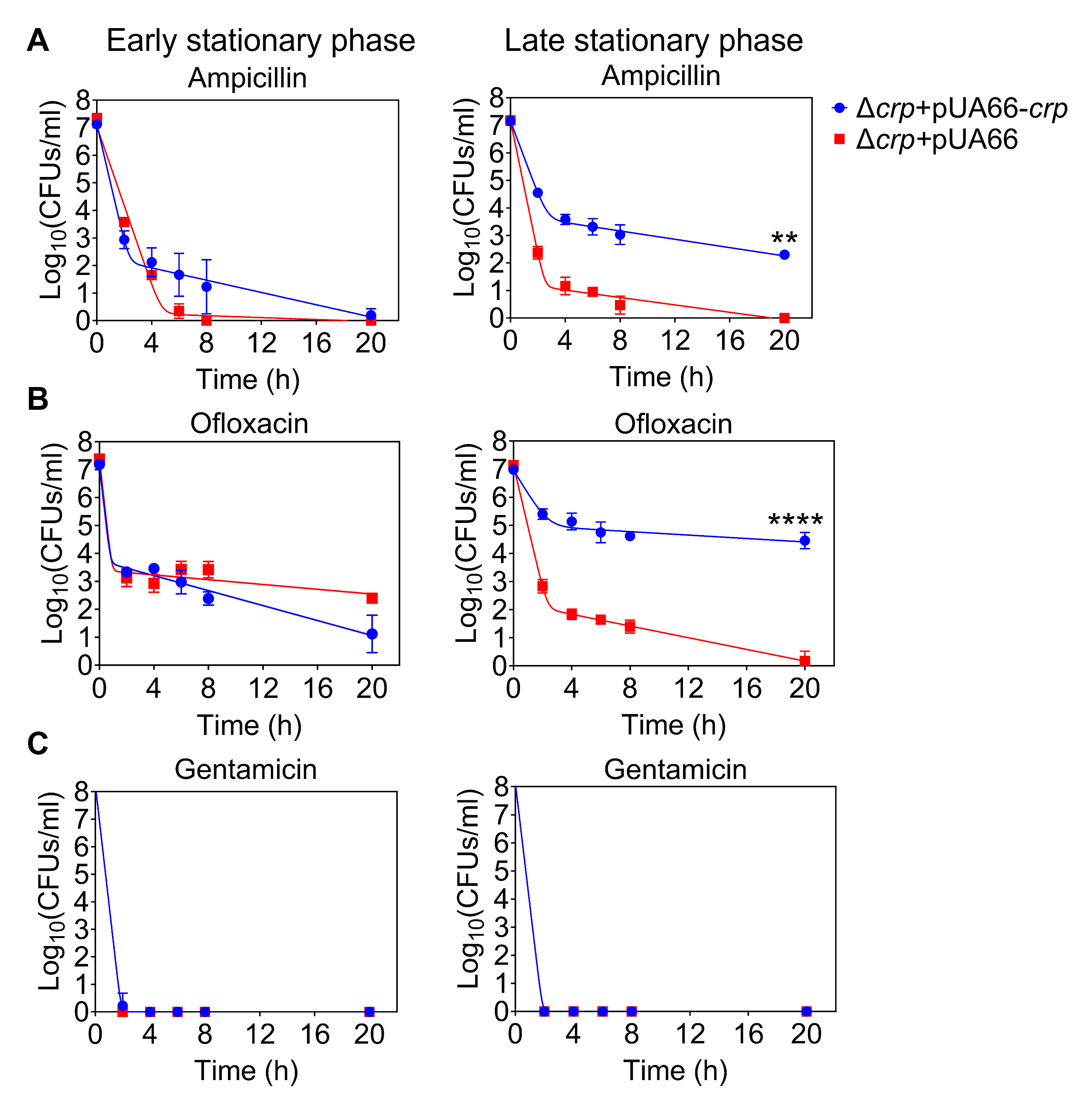

Supplement: Supplement 5 — Figure 1–figure supplement 5. Persister levels of cells carrying the Crp expression system. Cells at early (t=5 h) and late (t=24 h) stationary phases were transferred to fresh media with antibiotics for persister cell quantification. At time points 0, 2, 6, 8, and 20 h, 1 mL of the treated culture underwent two washes with 1X PBS to remove antibiotics. It was then serially diluted and plated on an agar plate to count the CFUs. (A) Persister levels of ampicillin-treated culture (200 μg/mL). (B) Persister levels of ofloxacin-treated cultures (5 μg/mL). (C) Persister levels of gentamicin-treated culture (50 μg/mL). n=4. Biphasic kill curves were generated using a non-linear model (see Materials and Methods). Statistical significance tests were conducted using F-statistics (**P < 0.01 and ****P < 0.0001). The data for each time point represent the mean value ± standard deviation. [file media-5.tif]

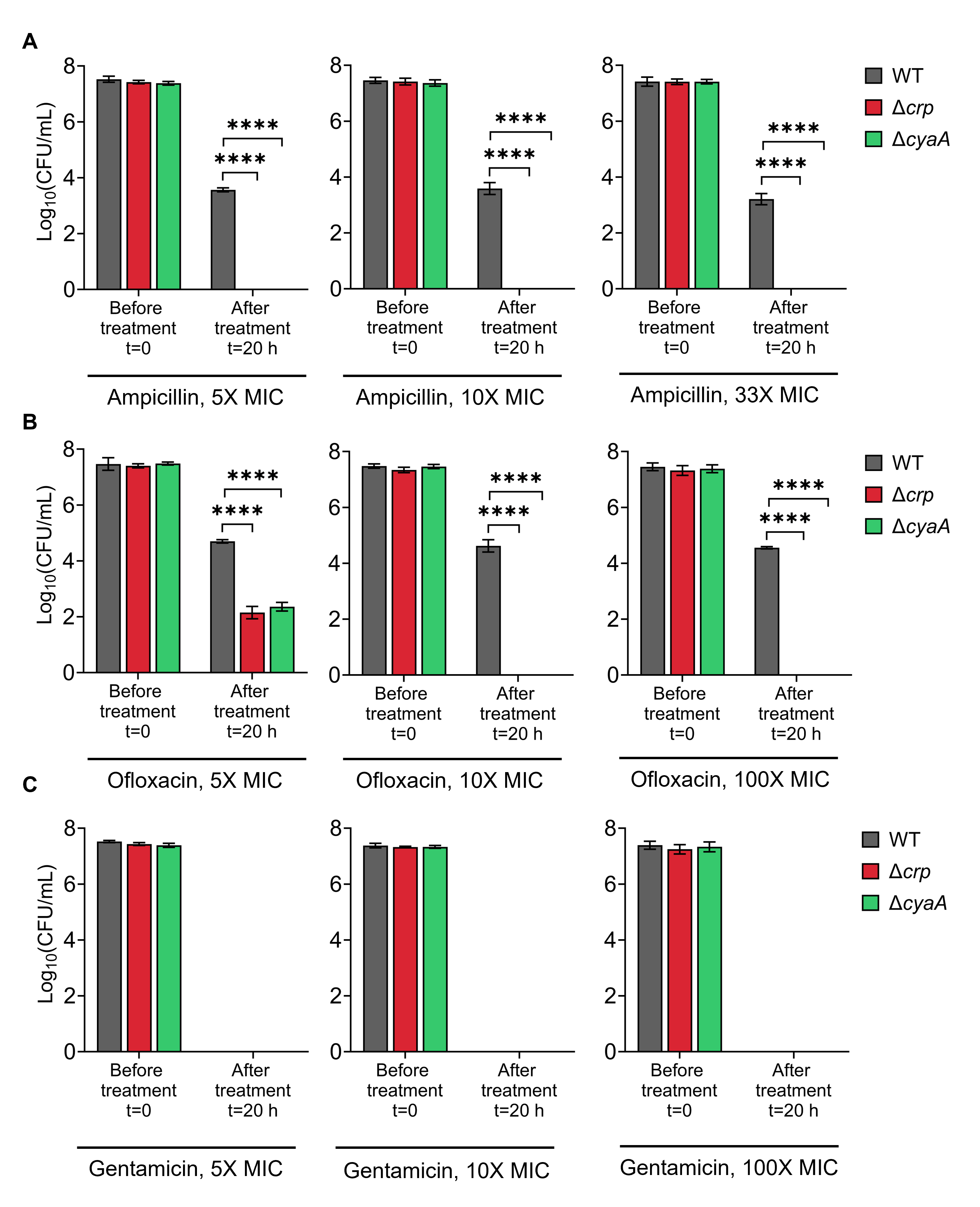

Supplement: Supplement 6 — Figure 1–figure supplement 6. Persister levels of E. coli K-12 MG1655 WT, Δcrp, and ΔcyaA strains at normalized antibiotic concentrations. Panels show survival following treatment with (A) ampicillin, (B) ofloxacin, and (C) gentamicin. After treatment, samples were washed six times with 1X PBS to minimize antibiotic carryover. Antibiotic concentrations were normalized to MICs to ensure valid comparisons across strains (The concentrations of 33× MIC for ampicillin and 100× MIC for ofloxacin and gentamicin match those used in Figure 1). n=4. Statistical significance was observed between control and mutant strains (****P < 0.0001, One-way ANOVA with Dunnett’s multiple comparisons test). The data for each time point represent the mean value ± standard deviation. [file media-6.tif]

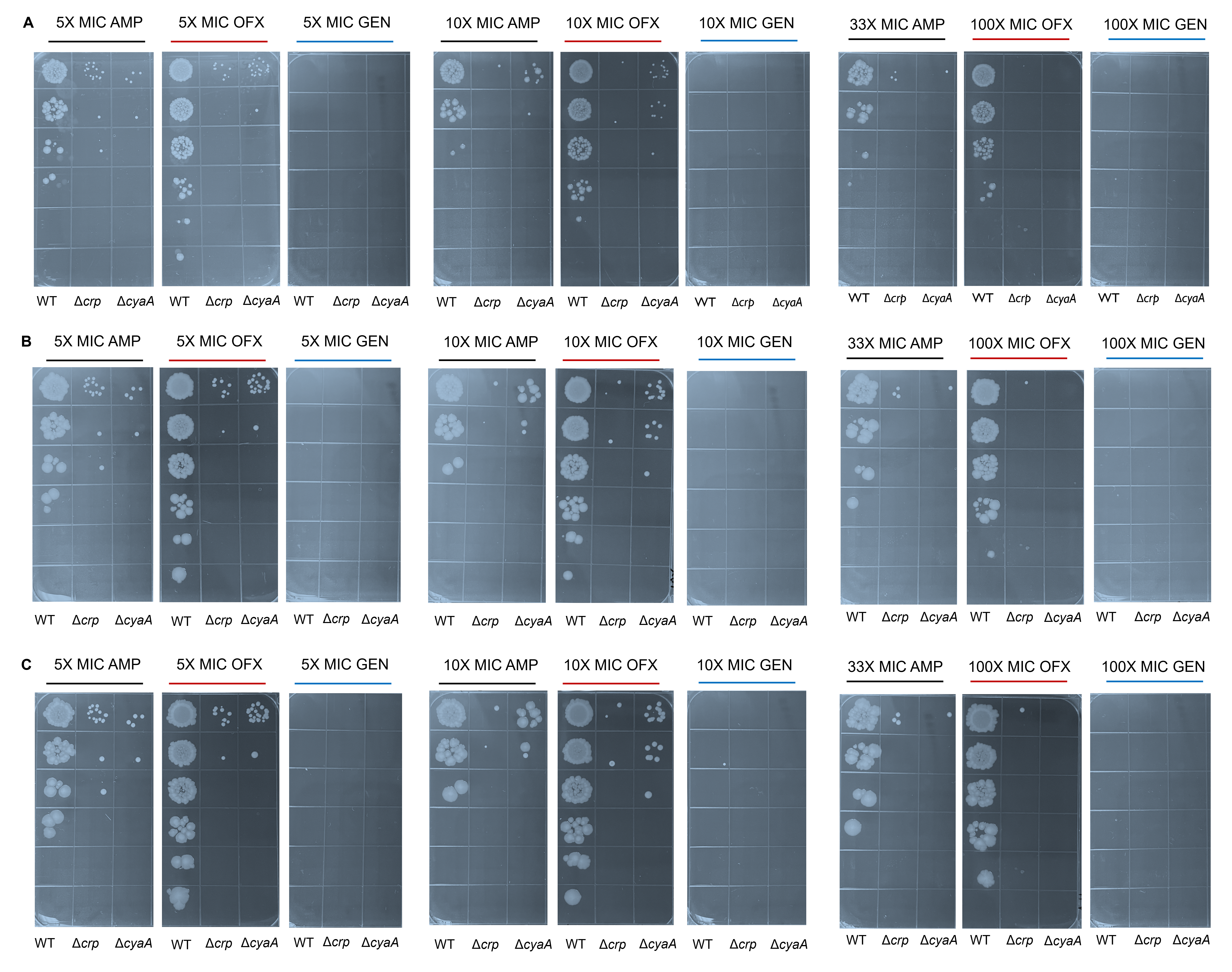

Supplement: Supplement 7 — Figure 1–figure supplement 7. Agar plates showing E. coli K-12 MG1655 WT, Δcrp, and ΔcyaA strains following treatment with antibiotics at normalized concentrations. After treatment, cells were washed and subjected to 10-fold serial dilutions, then plated on agar. Plates were incubated for (A) 16 h, (B) 48 h, and (C) 72 h to assess colony formation over time. The panel is a representative biological replicate. Consistent results were seen across all 3 biological replicates. [file media-7.tif]

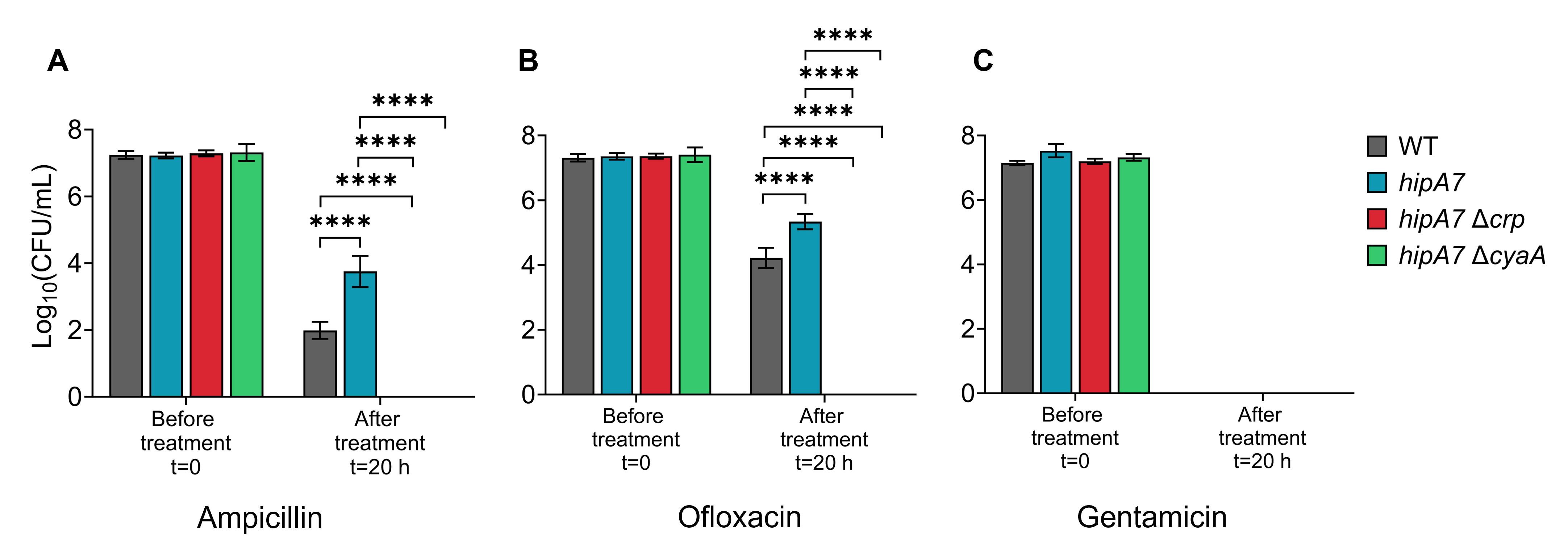

Supplement: Supplement 8 — Figure 1–figure supplement 8. Deletion of crp and cyaA reduces ampicillin and ofloxacin persistence in the hipA7 strain. Persistence levels were assessed by exposing E. coli K-12 MG1655 WT, hipA7, hipA7Δcrp, and hipA7ΔcyaA strains in the late stationary phase to the specified antibiotics, followed by CFU quantification at designated time points. (A) Ampicillin (200 μg/ml), (B) Ofloxacin (5 μg/ml) and (C) Gentamicin (50 μg/ml). n=4. Statistical significance was observed between control and mutant strains (****P < 0.0001, One-way ANOVA with Dunnett’s multiple comparisons test). The data for each time point represent the mean value ± standard deviation. [file media-8.tif]

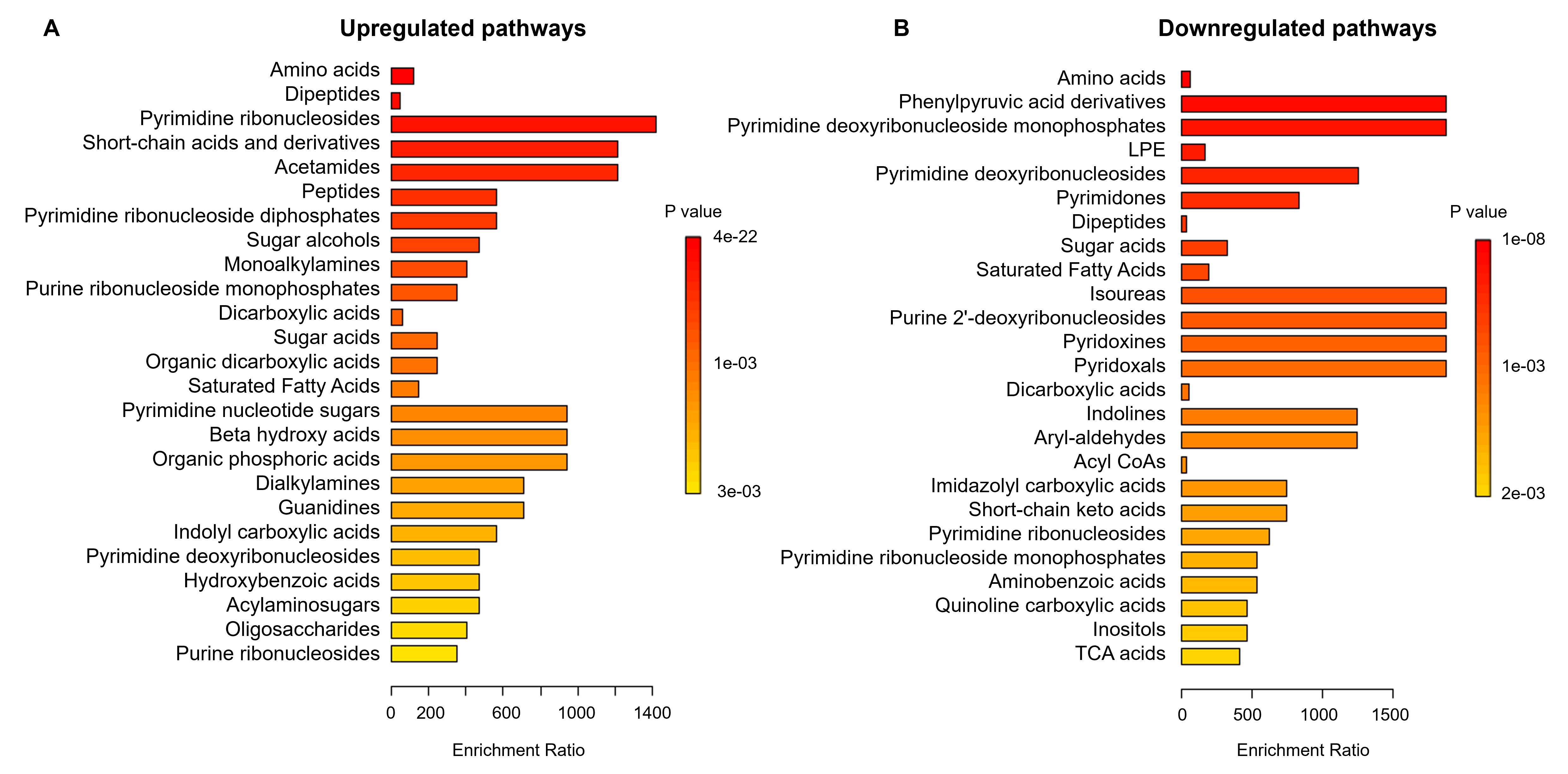

Supplement: Supplement 9 — Figure 2–figure supplement 1. The pathway enrichment analysis comparing WT and Δcrp strains during the early stationary phase cultures. The analysis was conducted using MetaboAnalyst, with a threshold ratio (Δcrp/WT) set at ≤ 0.5 for downregulation and ≥ 2 for upregulation. (A) Upregulated and (B) Downregulated pathways in the Δcrp strain compared to WT during the early stationary growth phase (ESP). [file media-9.tif]

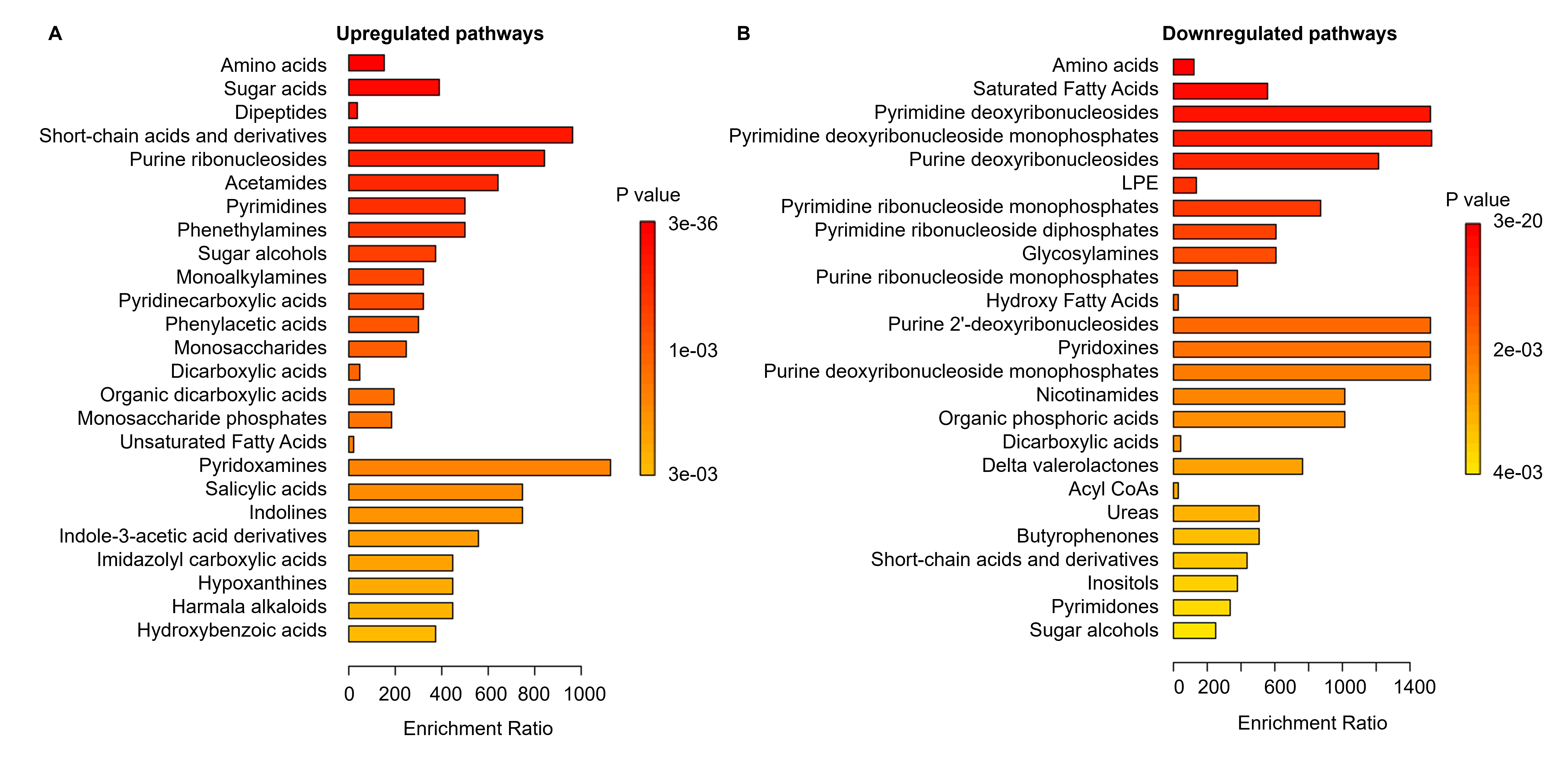

Supplement: Supplement 10 — Figure 2–figure supplement 2. The pathway enrichment analysis for the WT strain. The analysis was conducted using MetaboAnalyst, with a threshold ratio (LSP/ESP) set at ≤ 0.5 for downregulation and ≥ 2 for upregulation. (A) Upregulated and (B) Downregulated pathways of the WT strain in the late stationary growth phase (LSP) compared to the WT strain in the early stationary phase (ESP). [file media-10.tif]

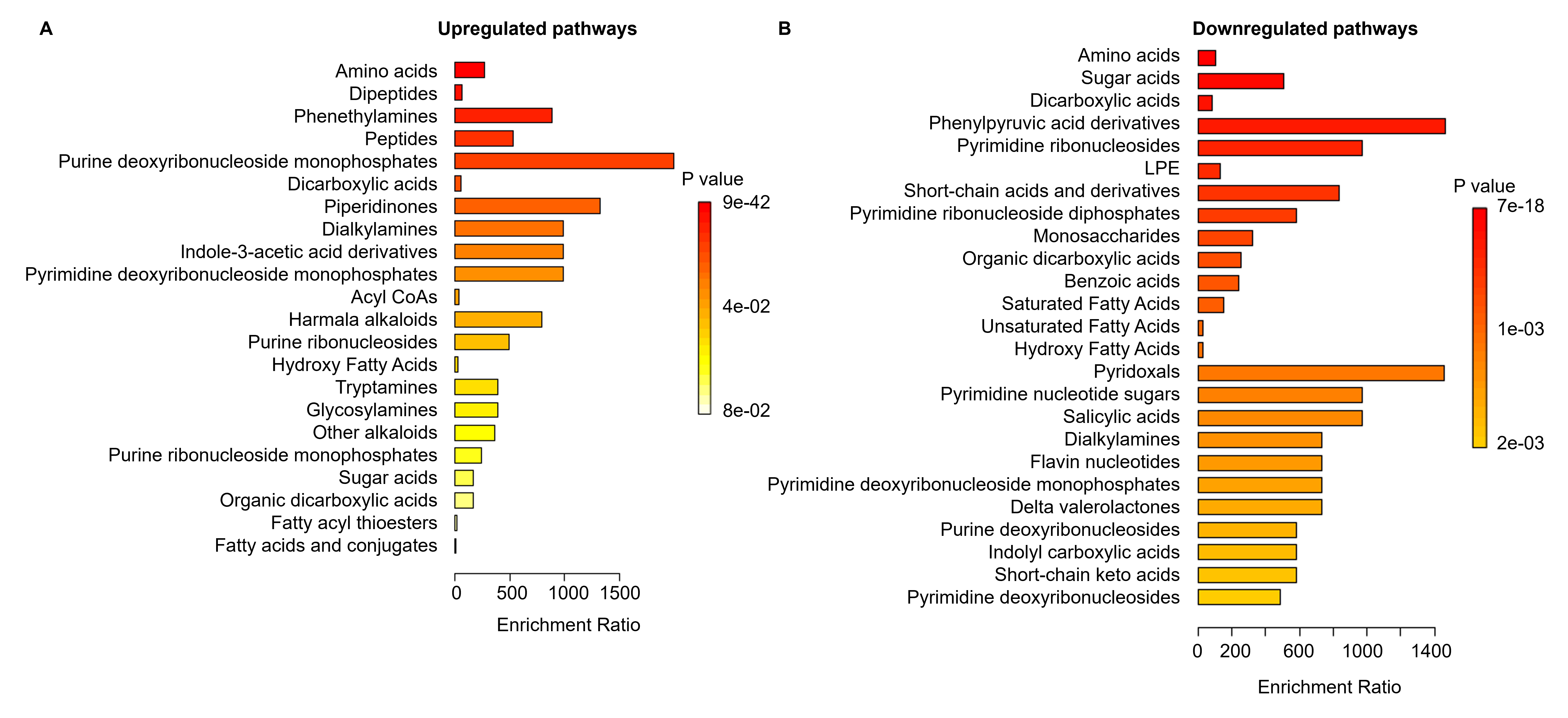

Supplement: Supplement 11 — Figure 2–figure supplement 3. The pathway enrichment analysis for the Δcrp strain. The analysis was conducted using MetaboAnalyst, with a threshold ratio (LSP/ESP) set at ≤ 0.5 for downregulation and ≥ 2 for upregulation. (A) Upregulated and (B) Downregulated pathways of the Δcrp strain in the late stationary growth phase (LSP) compared to the Δcrp strain in the early stationary phase (ESP). [file media-11.tif]

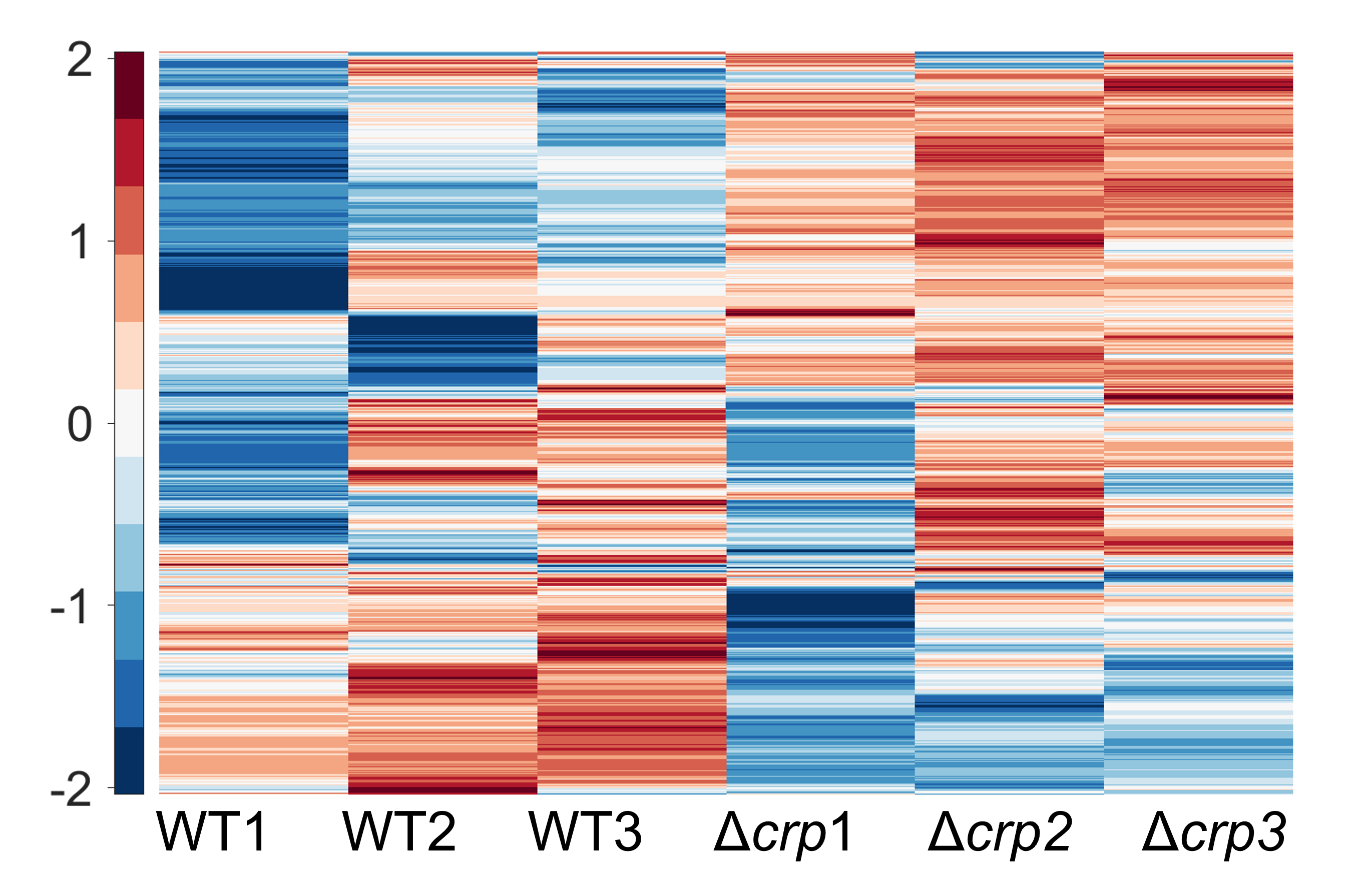

Supplement: Supplement 12 — Figure 3–figure supplement 1. The MS analysis of proteins from both WT and Δcrp strains at the late stationary phase. The proteomic data were subjected to unsupervised hierarchical clustering. Each column in the figure represents a biological replica. n=3. [file media-12.tif]

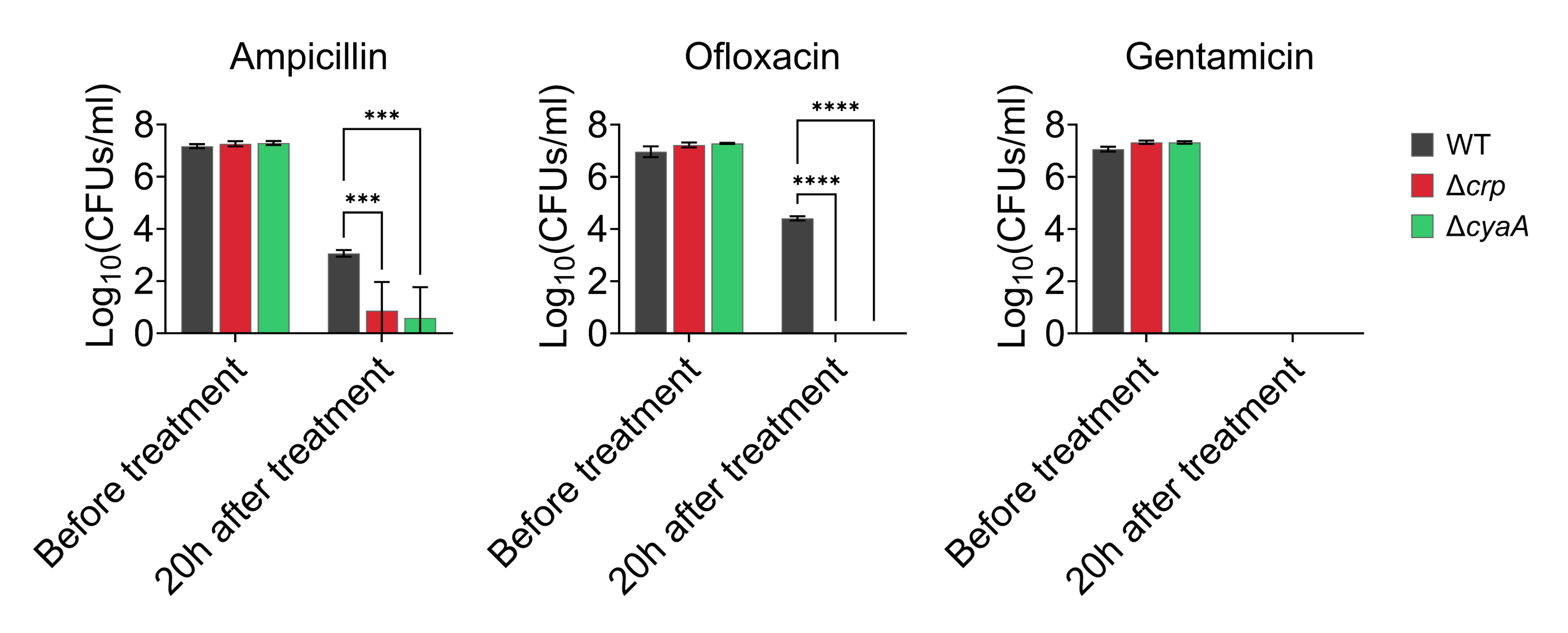

Supplement: Supplement 13 — Figure 4–figure supplement 1. Persister levels of E. coli WT, Δcrp, and ΔcyaA cells with the integrated mCherry expression system. Late stationary phase cultures (t=24 h) were transferred to fresh media and treated with ampicillin (200 μg/mL), ofloxacin (5 μg/mL), and gentamicin (50 μg/mL) for 20 hours. Subsequently, 1 mL of the treated culture underwent two washes with 1X PBS to remove antibiotics. It was then serially diluted and plated on an agar plate to count the CFUs. The levels of ofloxacin and gentamicin persisters in the mutant strains were below the limit of detection. n=4. Statistical significance was observed between control and mutant strains (***P < 0.001, ****P < 0.0001, Two-way ANOVA with Tukey’s multiple comparisons test). The data for each time point represent the mean value ± standard deviation. [file media-13.tif]

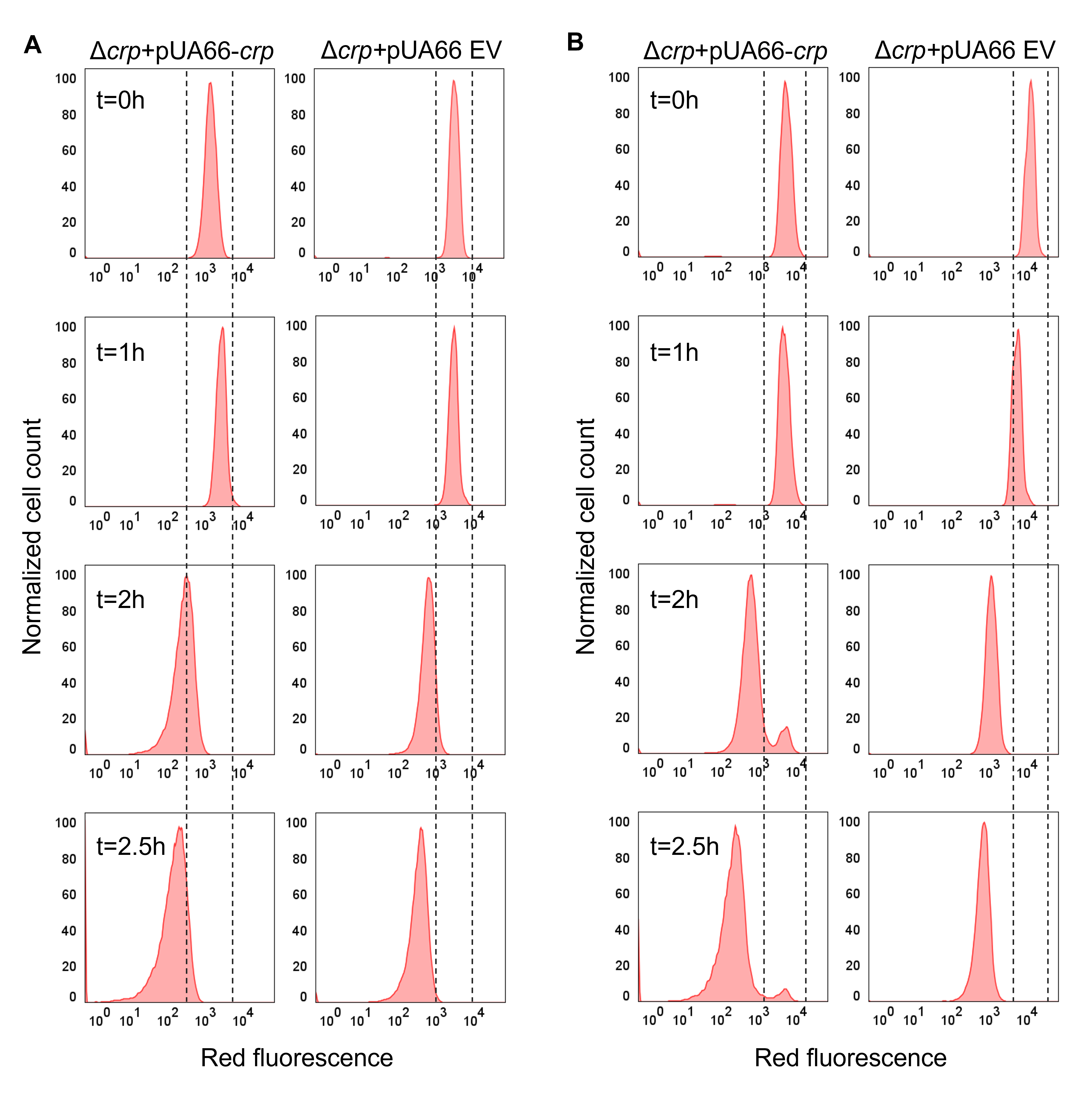

Supplement: Supplement 14 — Figure 4–figure supplement 2. Non-growing cell levels in the E. coli strain carrying the Crp expression system. (A, B) Flow cytometry histograms depict mCherry expression in Δcrp+pUA66-crp, and Δcrp+pUA66 EV at early (t=5 h) and late (t=24 h) stationary phases, respectively. Cells containing an IPTG-inducible mCherry expression system were cultivated with IPTG. After washing and dilution of early and late stationary phase cells in IPTG-free fresh media, fluorescence was tracked in non-growing and growing cells for 2.5 hours. The panel is a representative biological replicate. Consistent results were seen across all 3 biological replicates. [file media-14.tif]

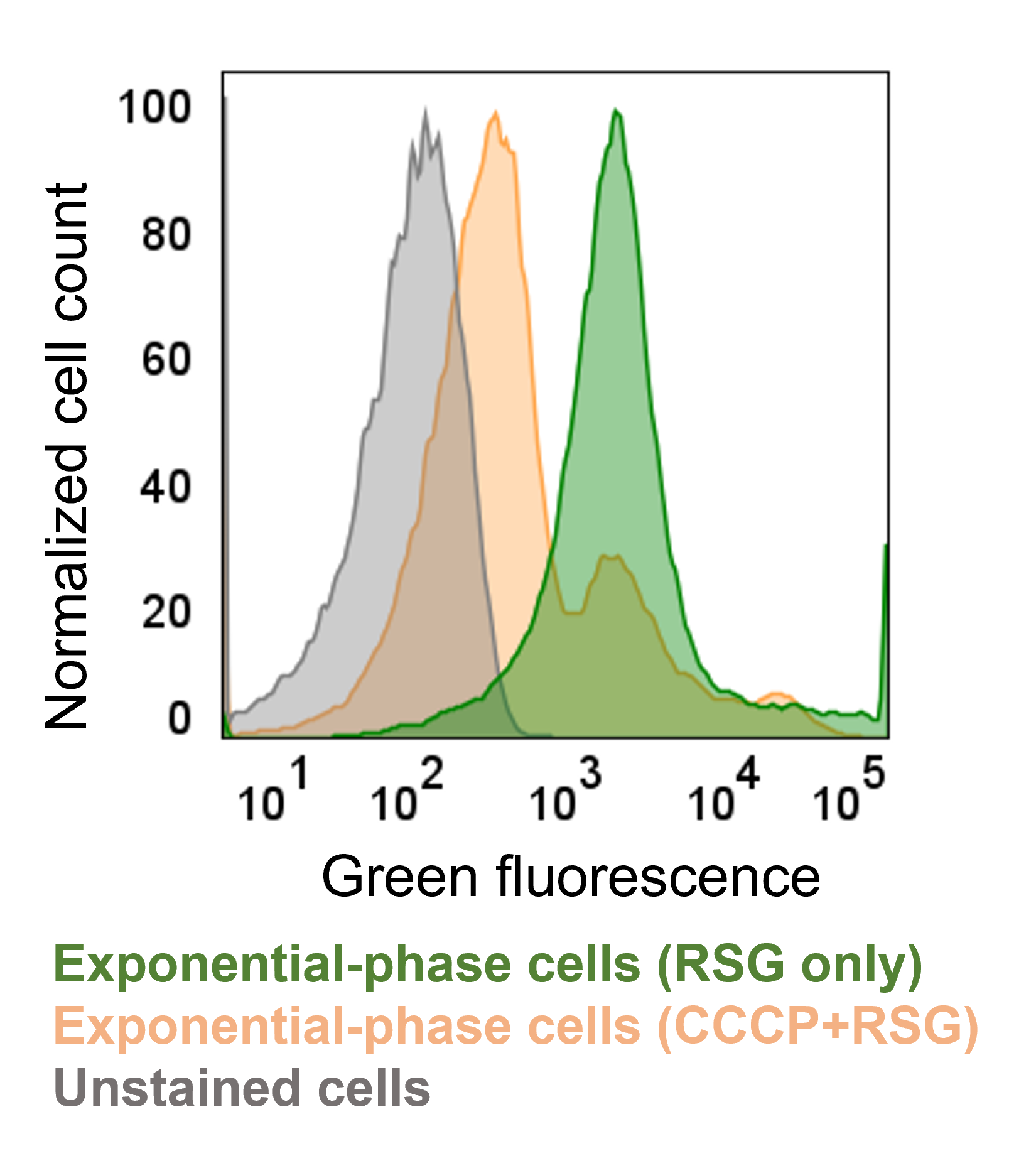

Supplement: Supplement 15 — Figure 5–figure supplement 1. RSG staining control for bacterial metabolic activities. Exponential phase (t=3 h) cells were stained with 1 μM RSG for 10 minutes at 37°C before analyzing by flow cytometry. Unstained cells and cells treated with 20 μM CCCP + 1 μM RSG were used as control. CCCP was expected to reduce cellular redox activities. The panel is a representative biological replicate. Consistent results were seen across all 3 biological replicates. [file media-15.tif]

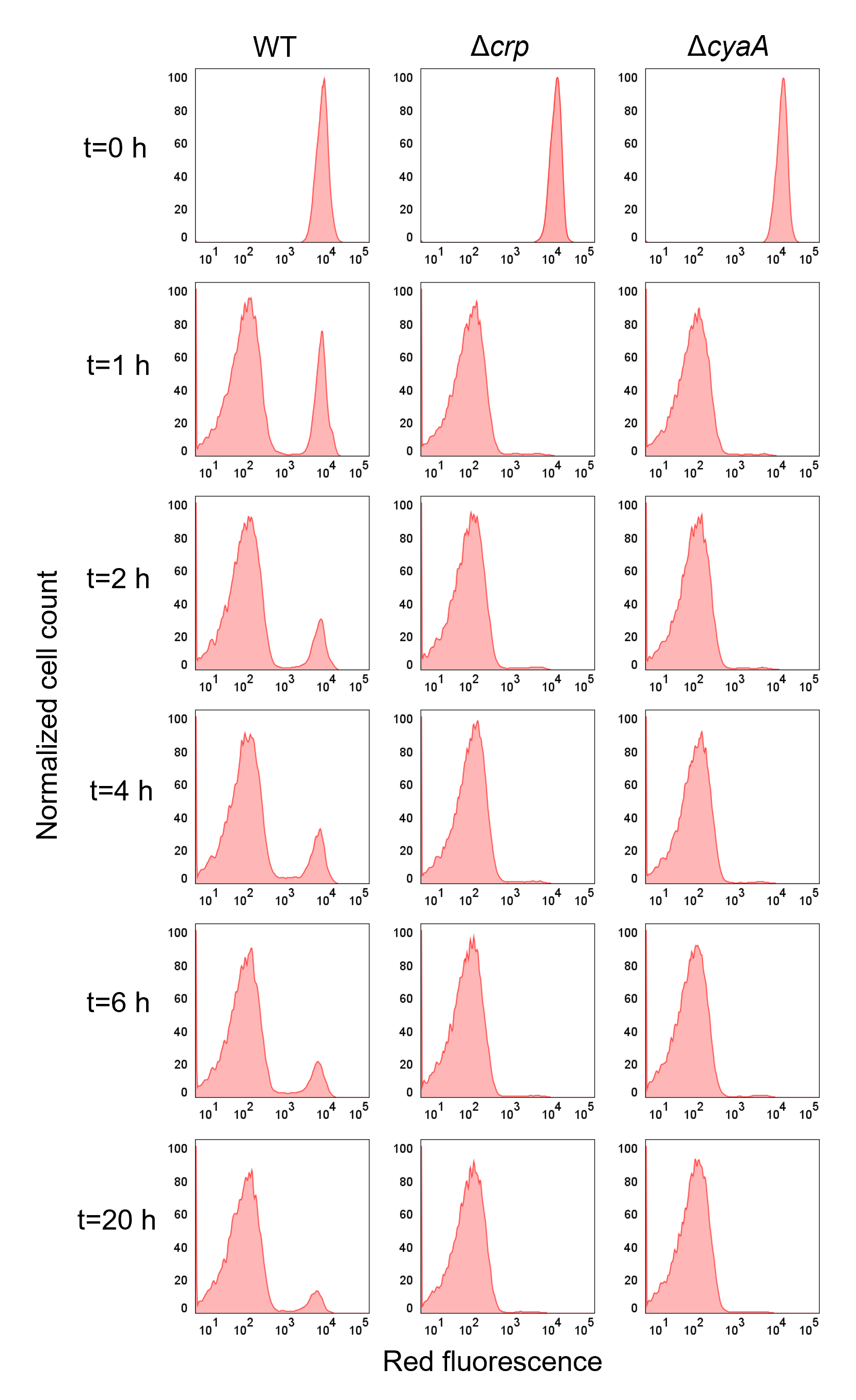

Supplement: Supplement 16 — Figure 5–figure supplement 2. Intact (non-lysed) cell levels of E. coli WT, Δcrp, and ΔcyaA cells with the integrated mCherry expression system. mCherry positive cells were diluted into fresh media and treated with ampicillin (200 μg/mL) for 20 hours. Flow cytometry was used to measure the red fluorescence of intact surviving cells at time points t=0, 1, 2, 4, 6 and 20 h. A representative biological replicate is shown, with consistent results across all 3 replicates. [file media-16.tif]

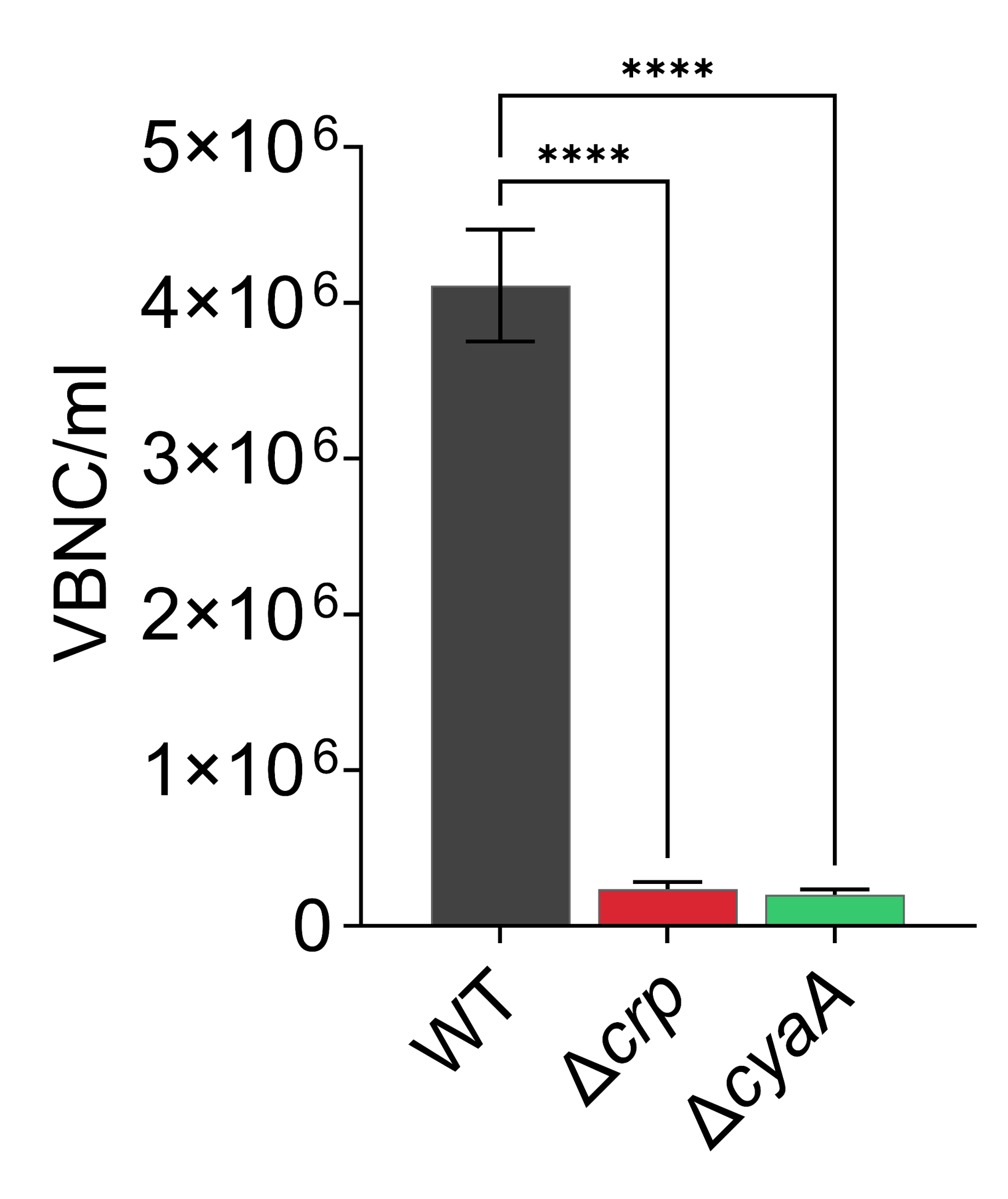

Supplement: Supplement 17 — Figure 5–figure supplement 3. VBNC levels of E. coli WT, Δcrp, and ΔcyaA cells with the integrated mCherry expression system. Flow cytometry was employed to quantify intact surviving cells. Persister cells were quantified by plating the cells on agar media. Viable but nonculturable (VBNC) cells were enumerated by subtracting persister levels from the intact cell levels. n=4. Statistical significance was observed between control and mutant strains (****P < 0.0001, One-way ANOVA using Dunnett’s multiple comparisons test). The data for each time point represent the mean value ± standard deviation. [file media-17.tif]

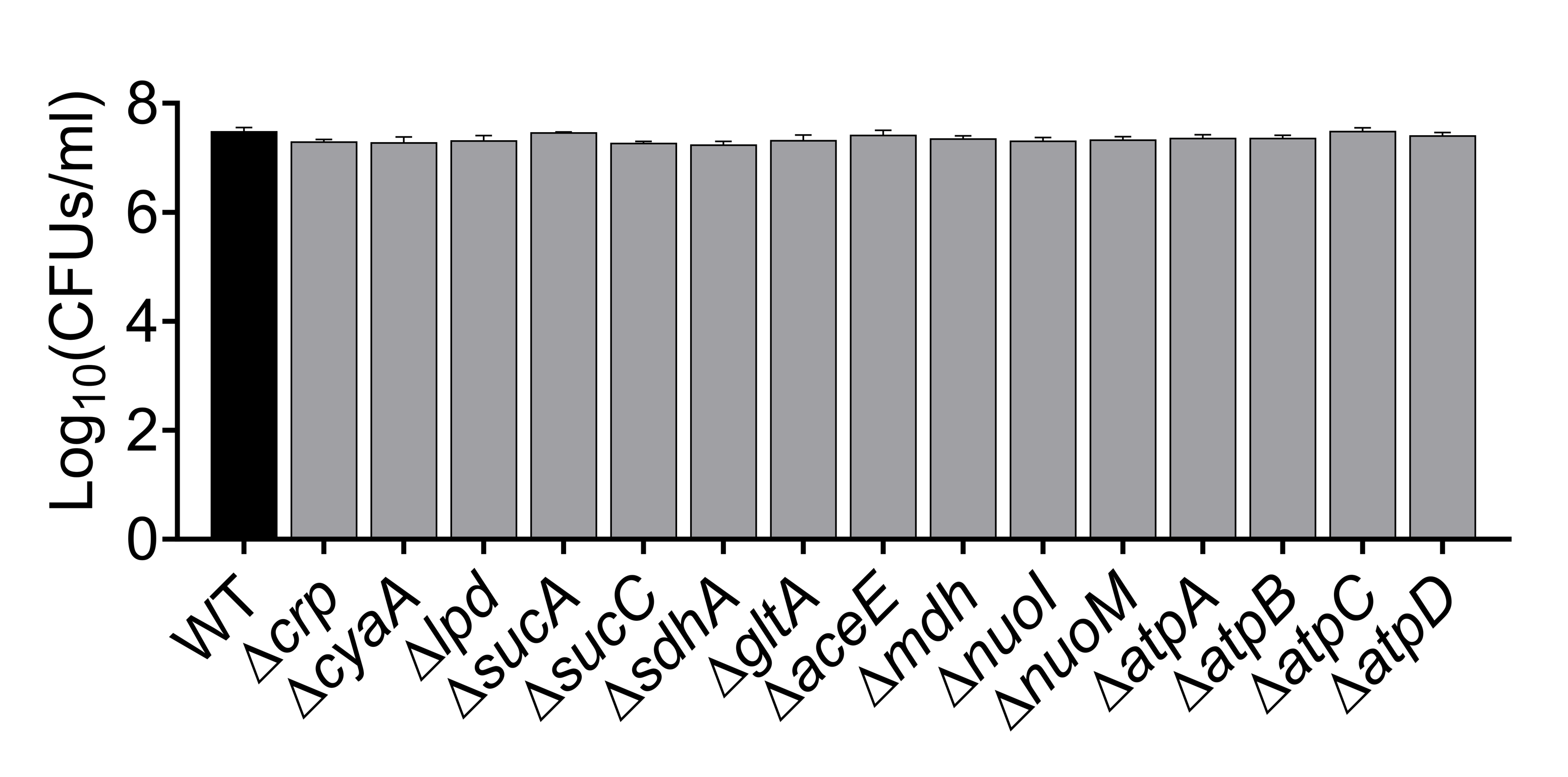

Supplement: Supplement 18 — Figure 5–figure supplement 4. Cell counts of E. coli K-12 MG1655 WT and mutant strains using flow cytometry at late stationary phase. Cells were diluted 100-fold into 1 mL of 1X PBS. n=4. The data for each time point represent the mean value ± standard deviation. [file media-18.tif]
