# Supplementary material for: UNRAVELING CRP/cAMP-MEDIATED METABOLIC REGULATION IN ESCHERICHIA COLI PERSISTER CELLS
Source: bioRxiv. 2025 Apr 8:2024.06.10.598332. Originally published 2024 Jun 10. Preprint. [Version 2] doi: 10.1101/2024.06.10.598332 (PMC11195080; doi:10.1101/2024.06.10.598332)
Supplement: Supplement 22 — Supplementary File 1. MIC of antibiotics and concentrations of bactericidal antibiotics used in persister assays. [file media-22.docx]

**Supplementary File 1. MIC of antibiotics and concentrations of bactericidal antibiotics used in persister assays.**

|  | Concentration (μg/mL) | | |
| --- | --- | --- | --- |
| **Bacterial Strains** | **Ampicillin** | **Ofloxacin** | **Gentamicin** |
| MIC of *E. coli* K-12 MG1655 Wild Type | 3-4 | 0.032-0.047 | 0.19-0.25 |
| MIC of *E. coli* K-12 MG1655 Δ*crp* | 4-6 | 0.064-0.094 | 3-4 |
| MIC of *E. coli* K-12 MG1655 Δ*cyaA* | 6-8 | 0.064-0.094 | 3-4 |
| Persister Assay Concentration | 200 | 5 | 50 |
