# Supplementary material for: UNRAVELING CRP/cAMP-MEDIATED METABOLIC REGULATION IN ESCHERICHIA COLI PERSISTER CELLS
Source: bioRxiv. 2025 Apr 8:2024.06.10.598332. Originally published 2024 Jun 10. Preprint. [Version 2] doi: 10.1101/2024.06.10.598332 (PMC11195080; doi:10.1101/2024.06.10.598332)

**Supplementary File 3. Analyzed proteomics data.**

**Supplementary File 3A. The pathway analysis for the upregulated proteins in the Δ*crp* strain compared to WT.** This analysis integrates statistical analysis across the entire genome and includes various functional pathway classification frameworks such as Gene Ontology annotations, KEGG pathways, and Uniprot keywords. Count in Network: The first number indicates how many proteins in our network are annotated with a particular term. The second number indicates how many proteins in total (in our network and in the background) have this term assigned. Strength: Log_10_(observed/expected). This measure describes how large the enrichment effect is. It’s the ratio between i) the number of proteins in our network that are annotated with a term and ii) the number of proteins that we expect to be annotated with this term in a random network of the same size. False Discovery Rate: This measure describes how significant the enrichment is. Shown are p-values corrected for multiple testing within each category using the Benjamini–Hochberg procedure.


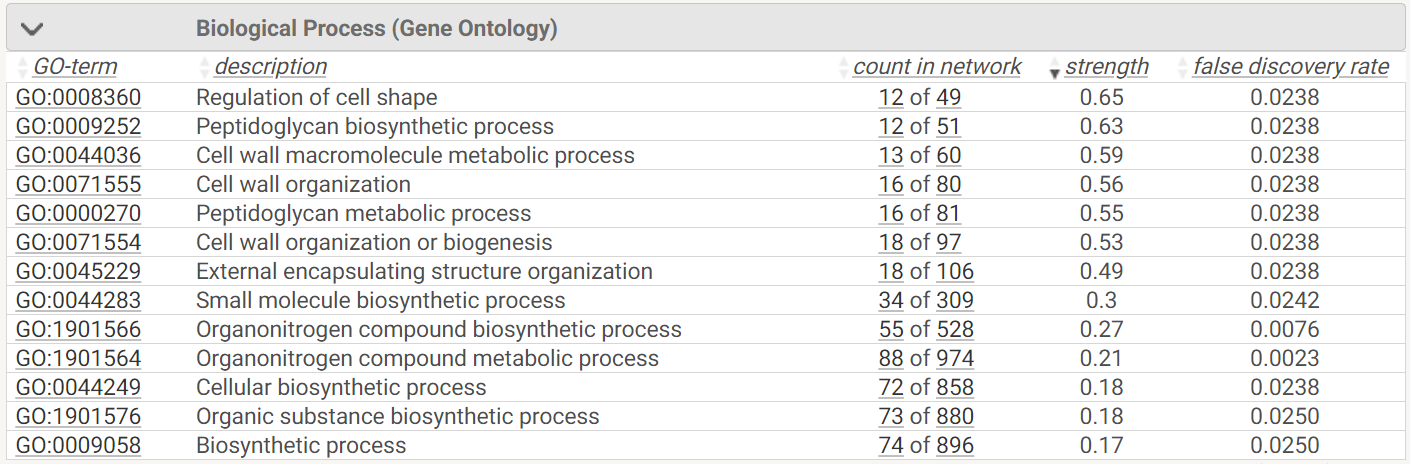


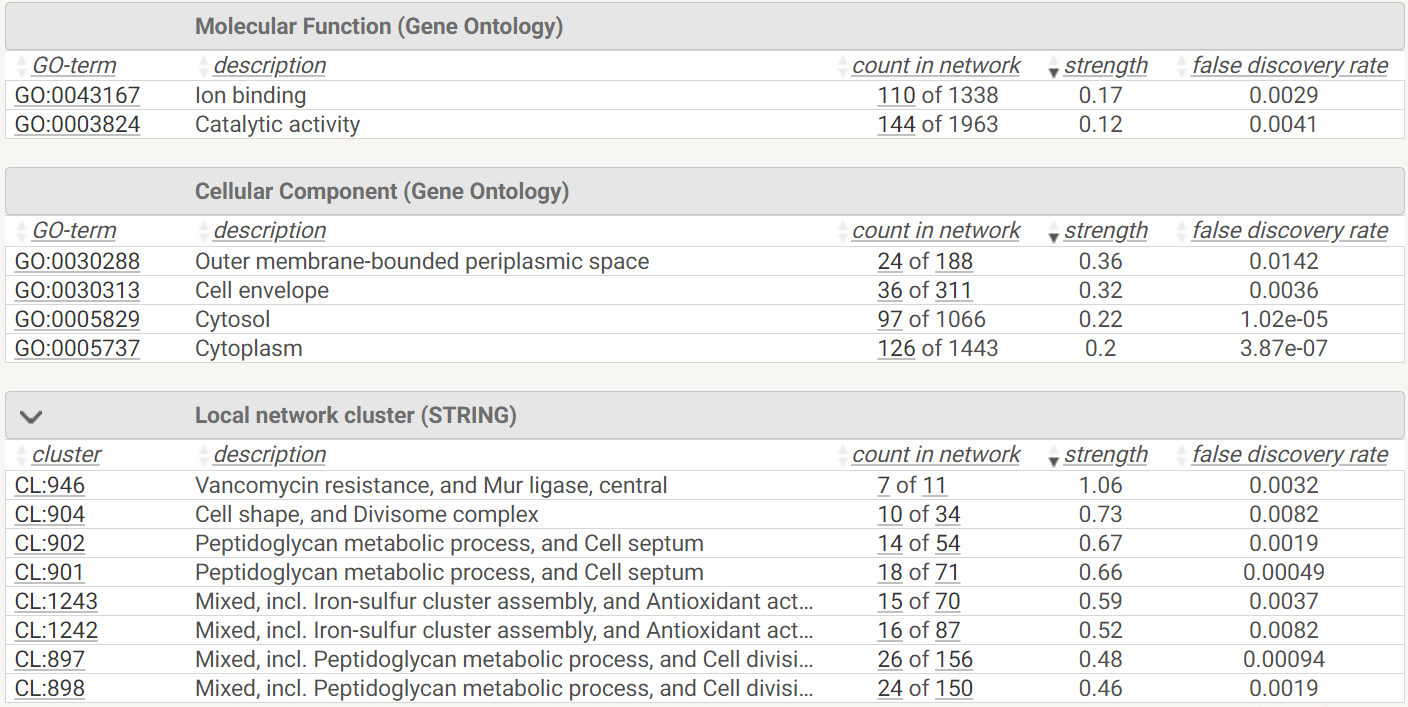


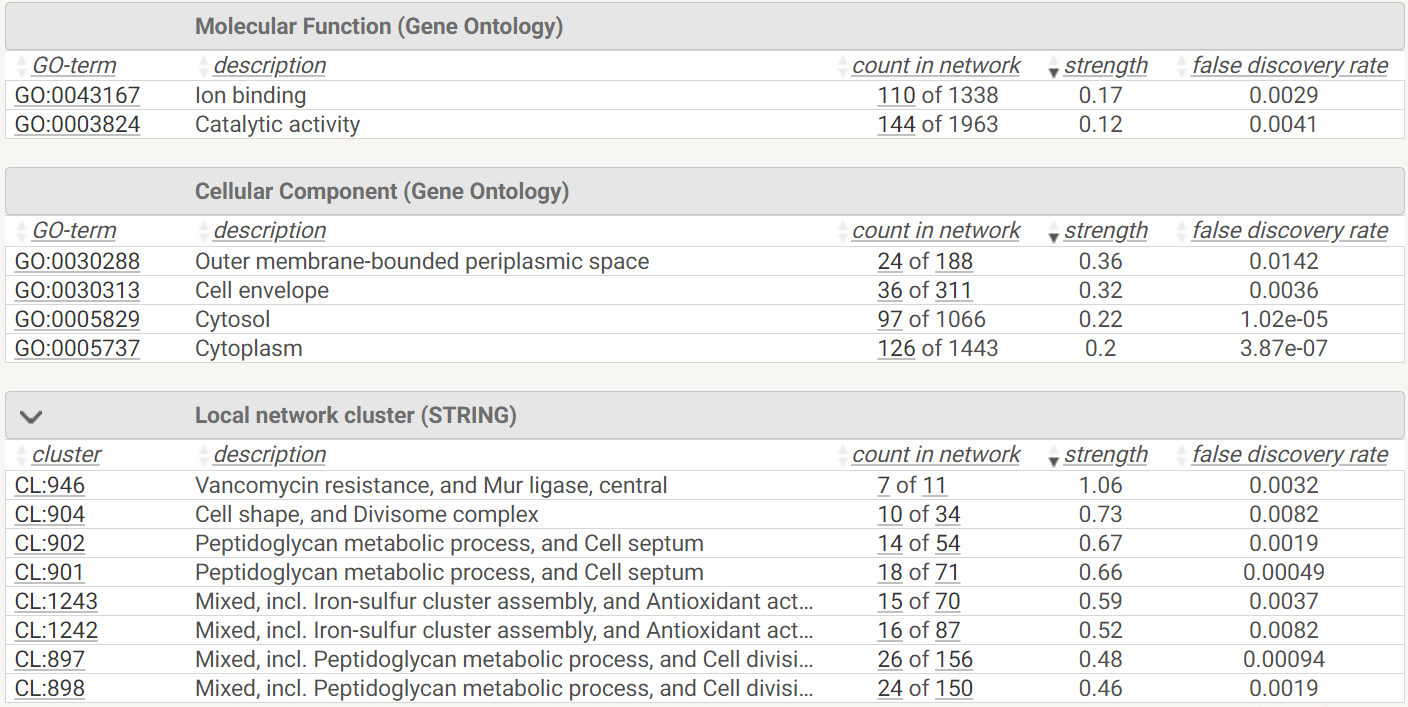


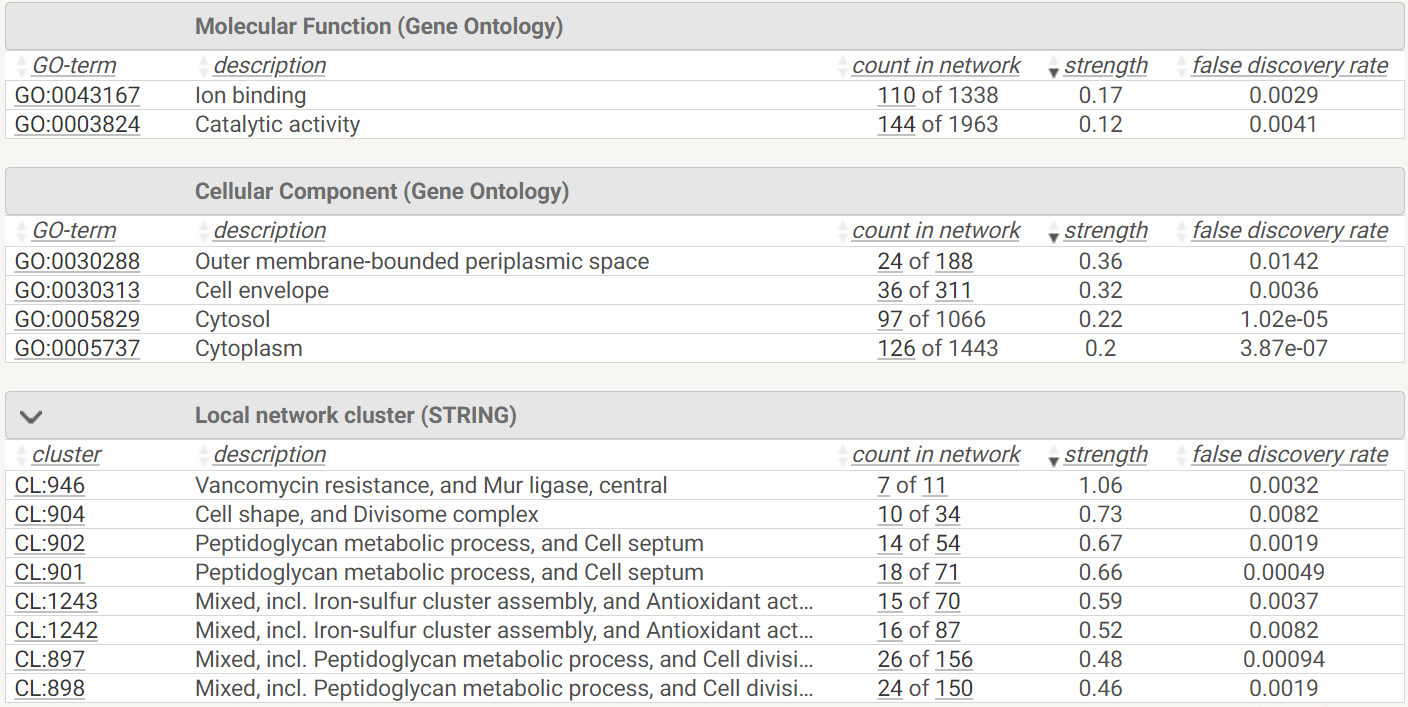


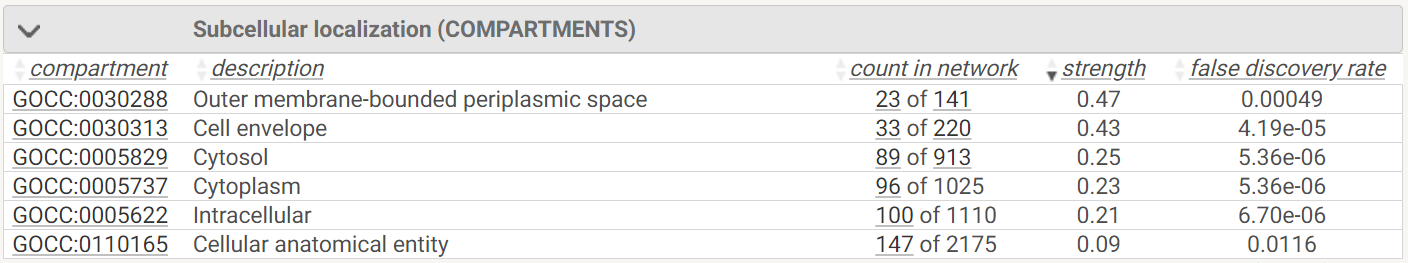


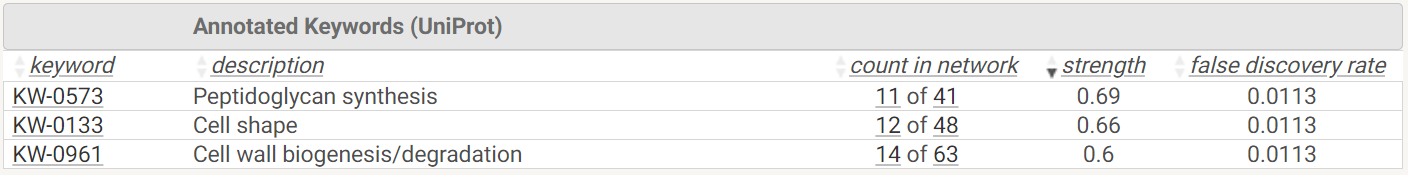


**Supplementary File 3B.** **The pathway analysis for the downregulated proteins in the Δ*crp* strain compared to WT.** See Supplementary File 3A for details.


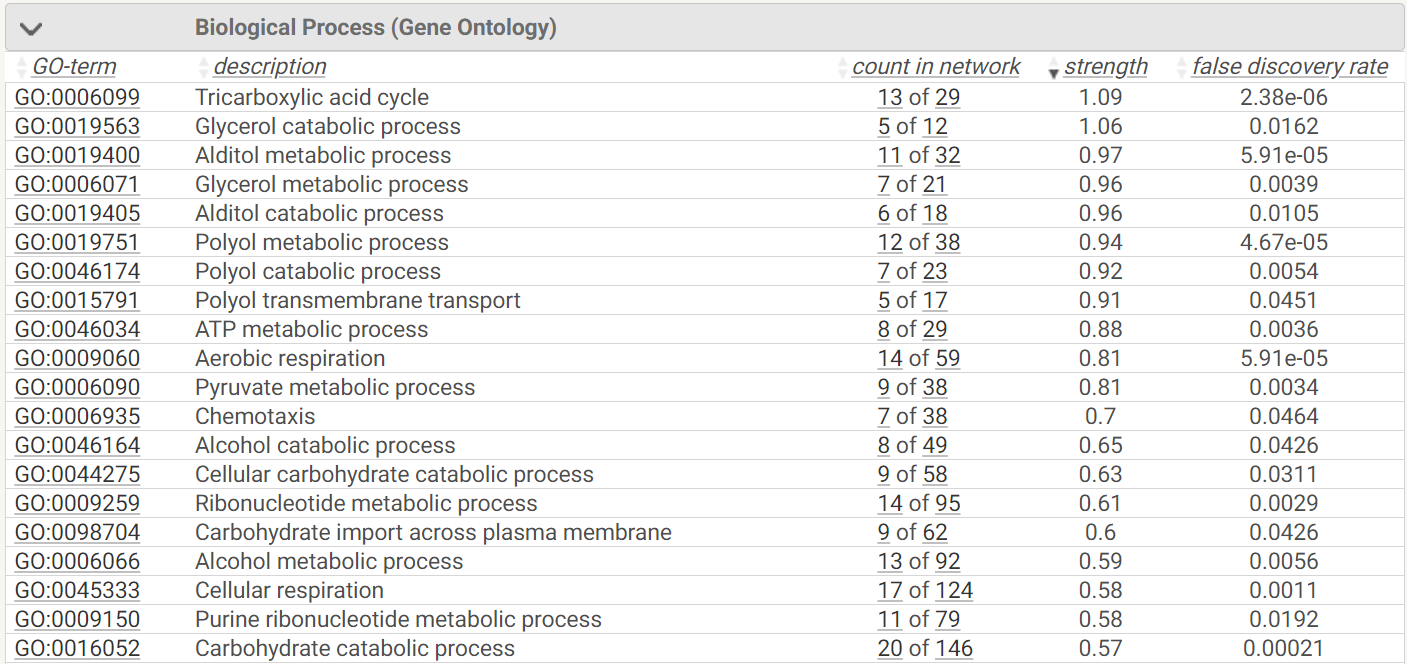


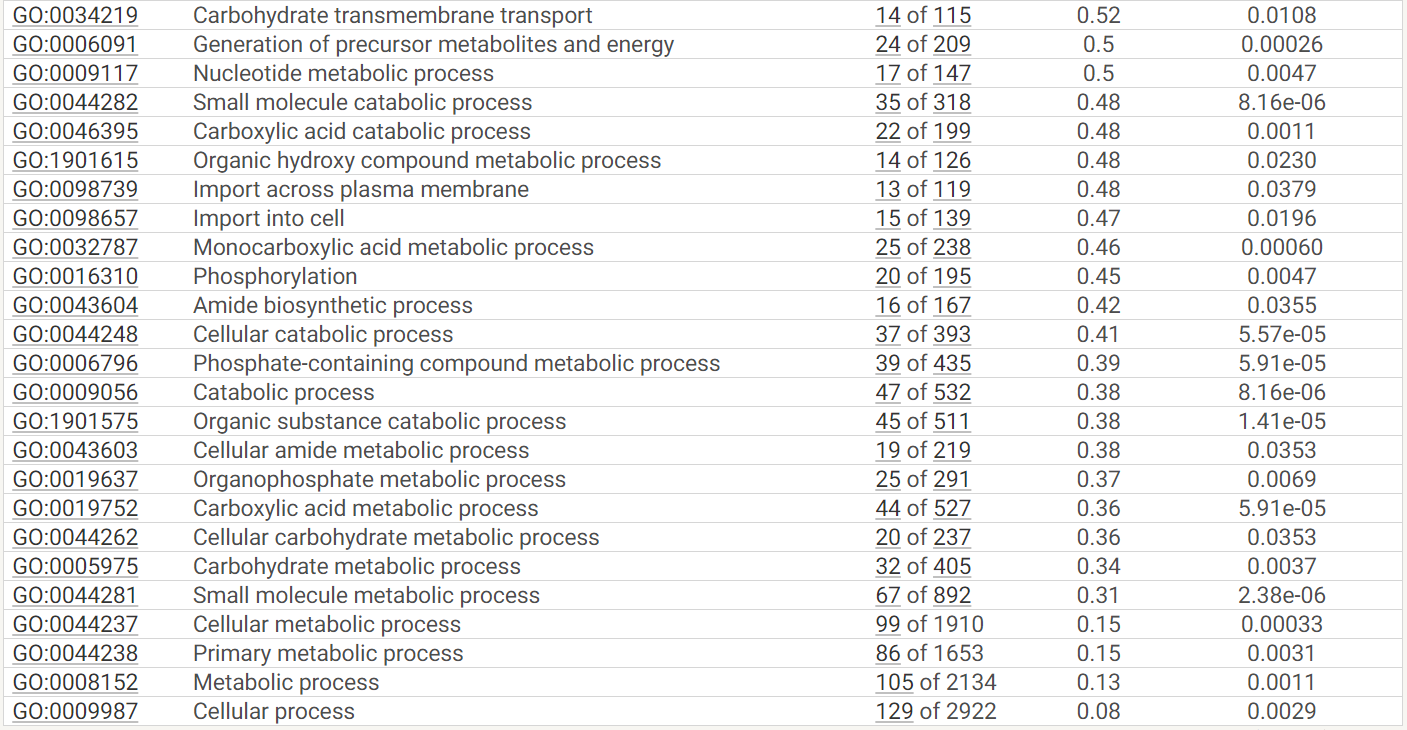


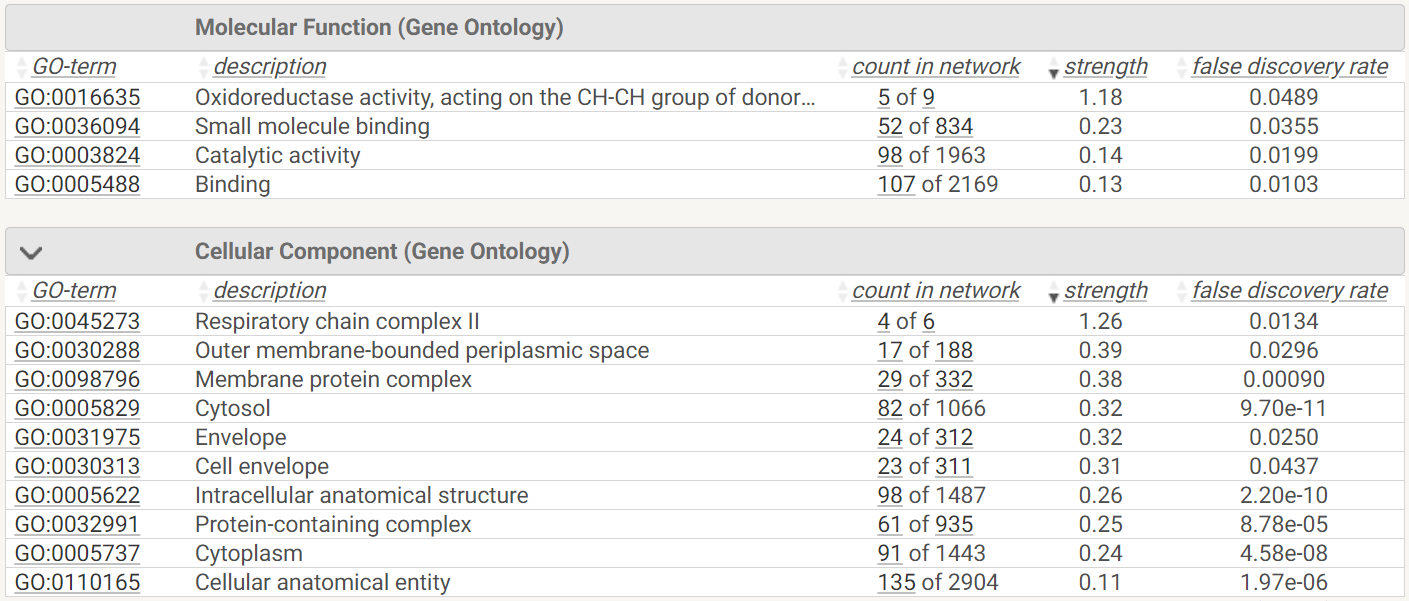


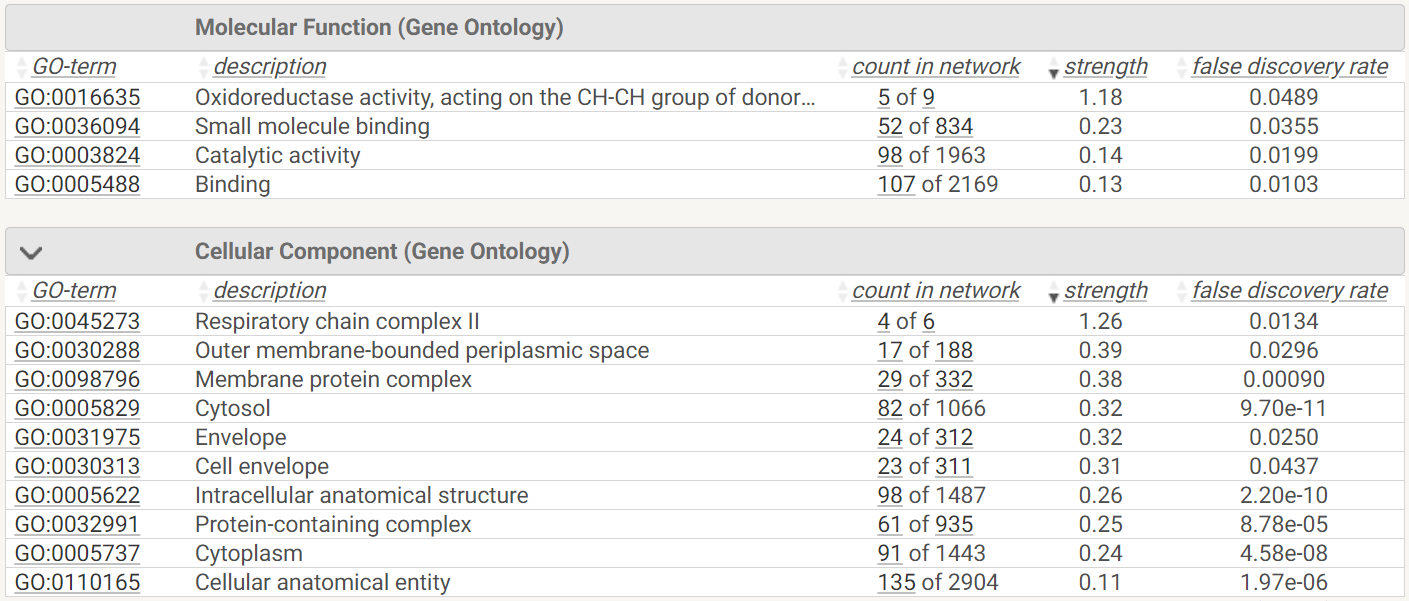


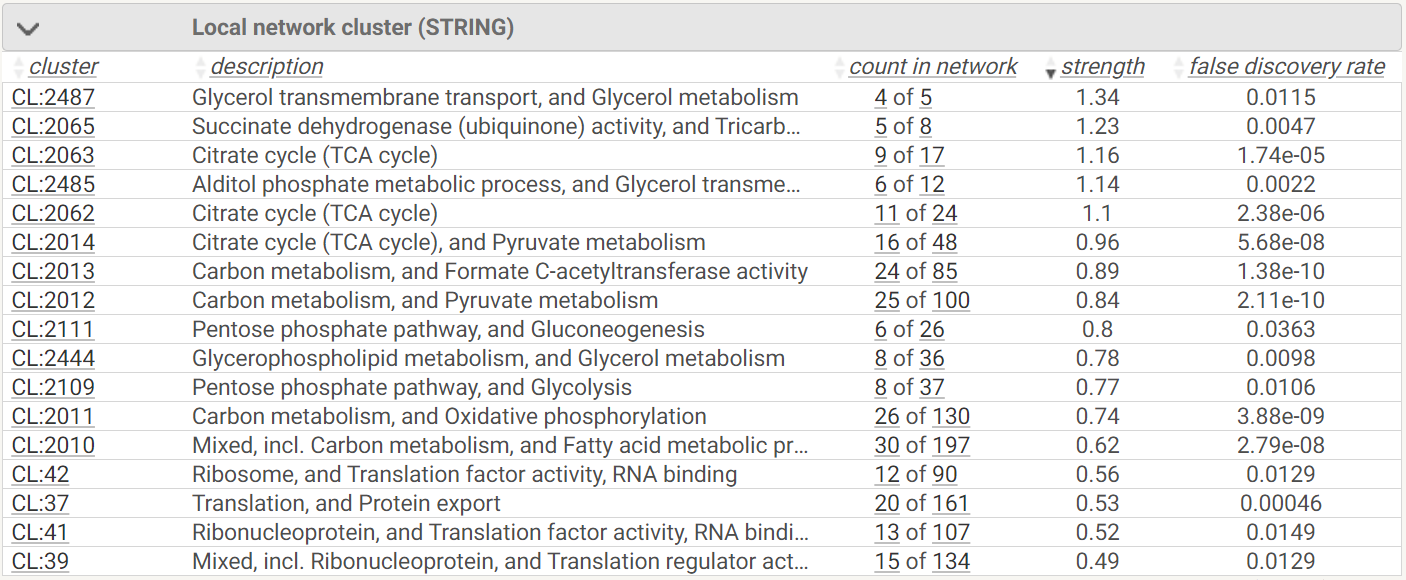


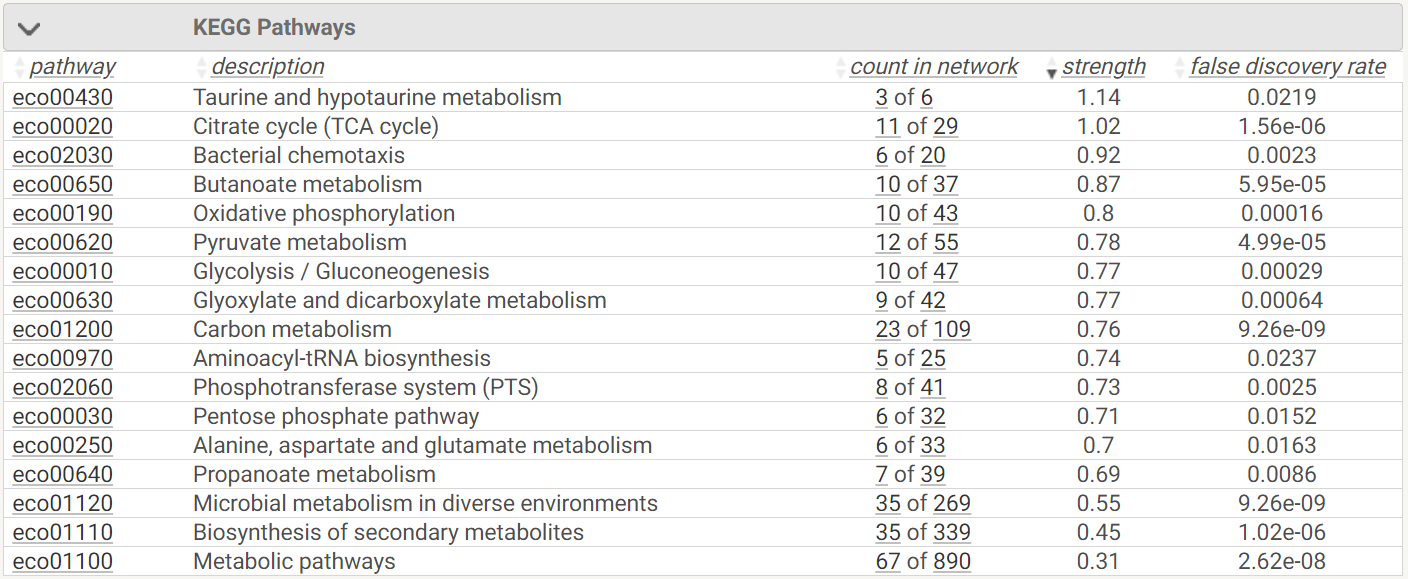


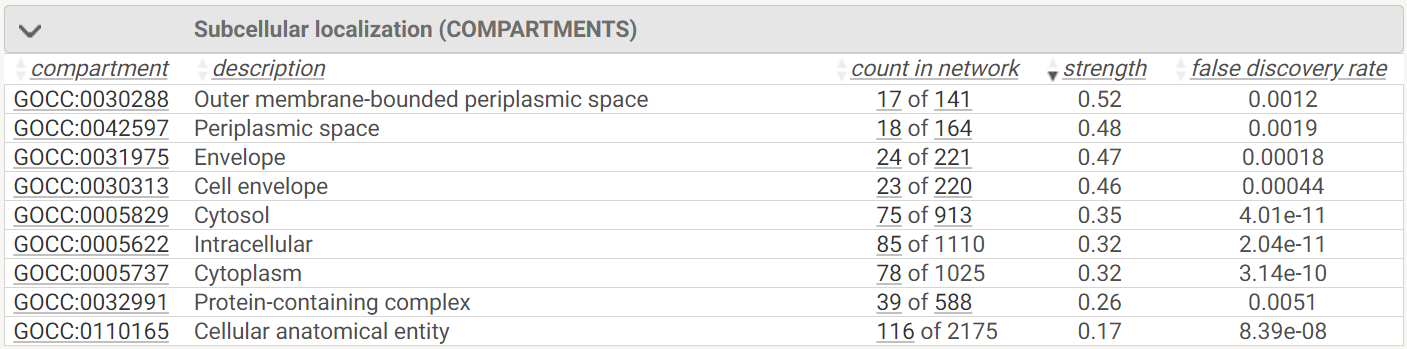


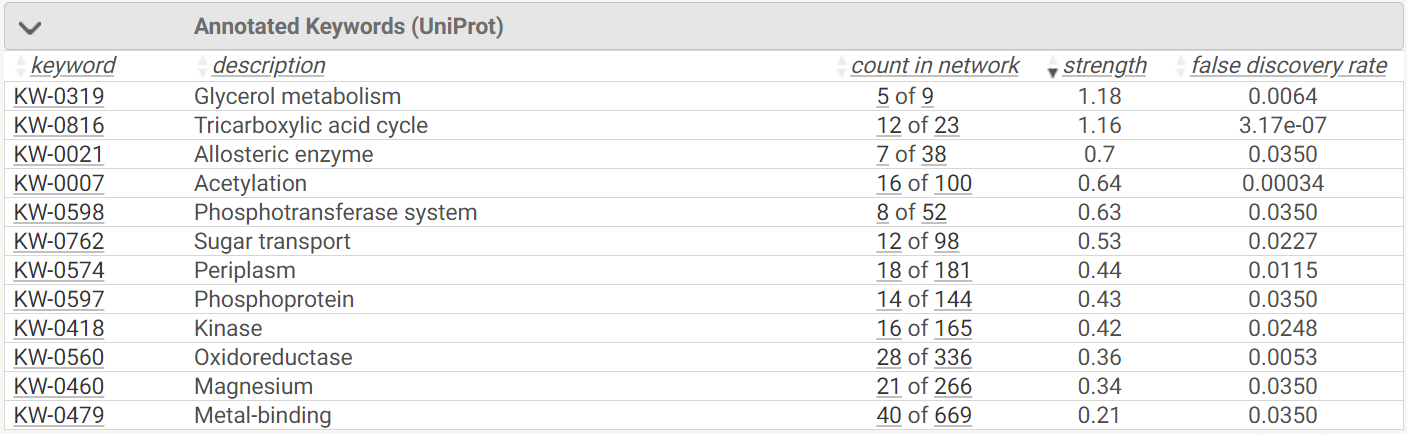

Supplement: Supplement 24 — Supplementary File 3. Analyzed proteomics data. [file media-24.docx]
